# Lipid Composition Determines Hybrid Nanoparticle Selectivity: Beyond Membrane Mimicry in Cancer Targeting

**DOI:** 10.1021/acs.nanolett.6c00637

**Published:** 2026-05-07

**Authors:** L. Gonzalo Espinoza-Arcos, Matías Valdés-Peña, Juan J. de Pablo, Cristian Vilos, Ricardo A. Zamora, Riccardo Alessandri, Horacio Poblete

**Affiliations:** † Centro de Bioinformática, Simulación y Modelado (CBSM), Facultad de Ingeniería, Campus Talca, 28066 Universidad de Talca, Talca 3465548, Chile; ‡ Department of Chemical Engineering, 418666 KU Leuven, 3001 Leuven, Belgium; § Departments of Chemical and Biological Engineering, Computer Science, and Physics, Tandon School of Engineering, and Courant Institute of Mathematical Sciences, New York University, New York, New York 11201, United States; ∥ Laboratory of Nanomedicine and Targeted Delivery, Faculty of Medicine, Universidad de Talca, Talca 3465548, Chile; ⊥ Center for Nanomedicine, Diagnostic and Drug Development (ND3 Center), Universidad de Talca, Talca 3465548, Chile; # Center for Nanoscience and Nanotechnology (CEDENNA), Proyecto CIA250002, Manuel Rodríguez Sur 415, Santiago 8370179, Chile; ○ Vicerrectoría Académica, and Escuela de Ingeniería Civil en Bioinformática, Facultad de Ingeniería, Universidad de Talca, Talca 3465548, Chile

**Keywords:** Lipid-functionalized nanoparticles, Membrane selectivity, Coarse-grained molecular dynamics, Rational nanocarrier design

## Abstract

Lipid-functionalized hybrid nanoparticles (hNPs) are promising for selective cancer delivery, due to their tunable membrane interactions. Yet, whether mimicking target membrane composition enhances recognition, and which determinants govern selectivity remains unresolved. Using coarse-grained molecular dynamics, umbrella sampling simulations, and lipid-specific decomposition analyses on 40 membrane-hNP systems, we examine how individual lipid species govern hNP interactions with mammalian-like and tumor-like bilayers. Our results showed cholesterol acts as the dominant stabilizer, generating free-energy minima and driving remodeling in both bilayers. Conversely, zwitterionic lipids showed weakened interactions, limited insertion, suppressed exchange, and entropic penalties. Tumor-like membranes amplify cholesterol’s role in mediating hNP–membrane recognition, facilitating deeper insertion and lipid reorganization. Strikingly, composition-matched hNPs did not preferentially bind their corresponding membrane, whereas cholesterol-enriched formulations displayed increased affinity and selectivity. Thus, lipid-composition mimicry fails as a design principle for selective recognition. These findings provide a mechanistic basis for rational lipid selection, emphasizing complementarity over membrane mimicry.

## Graphical abstract

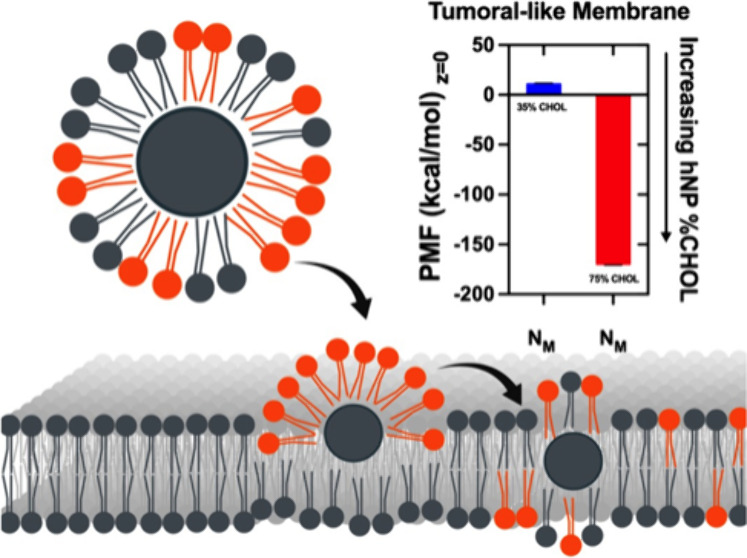

Targeted delivery to tumor sites remains a major challenge in nanoparticle-based drug delivery systems, as achieving precise accumulation of therapeutic cargo while minimizing off-target effects continues to limit clinical translation.^1,2^ Tumor heterogeneity, complex microenvironments, and biological barriers further hinder the selective accumulation of nanocarriers in malignant tissues, thereby reducing therapeutic efficacy.^3–6^ In this context, emerging nanotechnological approaches increasingly employ lipid-functionalized hybrid nanoparticles (hNPs) due to their versatility and tunable lipid compositions, which enable targeted interactions with biological membranes. Variations in lipid composition can modulate nanoparticle–membrane interactions, including binding, insertion, and recognition. However, it remains unclear whether these effects are driven primarily by overall compositional similarity to the target membrane or by the specific influence of individual lipid species.^7–9^

Cancer cells exhibit distinctive lipidomic signatures resulting from altered lipid metabolism. While mammalian membranes are typically enriched in phosphatidylcholine (PC), sphingomyelin (SM), and cholesterol (CHOL), tumor membranes display elevated levels of phosphatidylserine (PS) and phosphatidylethanolamine (PE), with reduced levels of PC, SM, and CHOL.^10–12^ In particular, CHOL content in mammalian membranes is approximately twice that of tumoral membranes, a difference that critically influences bilayer structure and dynamics.^13,14^ While CHOL accumulation increases membrane order and rigidity,^13,14^ SM–CHOL interactions drive the formation of lipid rafts.^15^ Nevertheless, despite these well-documented compositional differences, it is not yet clear how such lipidomic alterations translate into selective nanoparticle–membrane interactions, leaving the molecular determinants of recognition largely unresolved. Given pronounced lipidomic differences, lipid composition has long been considered a key determinant of nanoparticle selectivity.^16,17^ However, whether lipidome mimicry alone is sufficient to drive nanoparticle–membrane recognition remains unresolved. Emerging evidence suggests that selectivity is governed primarily by the nanoparticle’s intrinsic composition rather than by the membrane it is designed to mimic.^18,19^ In this context, it is essential to consider not only the overall lipid composition but also the specific contribution of individual lipids. CHOL has been described as promoting favorable interactions,^20,21^ whereas PC and PE often reduce affinity.^22^ However, these lipids are typically incorporated with polymers such as PEG, complicating the isolation of lipid-specific effects.^23,24^ Consequently, whether mammalian-like nanoparticles preferentially associate with mammalian membranes or instead shift toward tumor-associated membranes remains an open question, namely, whether hNP–membrane recognition is driven primarily by global lipidome similarity or by the contribution of individual lipid species.

Computational approaches, particularly coarse-grained (CG) simulations, provide powerful tools to investigate nanoparticle–membrane interactions. Molecular dynamics (MD) studies have shown that lipid composition can modulate nanoparticle binding, insertion, and orientation of hNPs in bilayers of varying complexity. MD simulations have also revealed molecular-level recognition mechanisms, such as cationic lipid nanoparticles exhibiting a two-step binding process that involves interfacial lipid reorganization between nanoparticle and membrane.^24–27^ Furthermore, CG models have been developed to simulate functionalized nanoparticles based on diverse polymers,^28^ as well as inorganic systems including fullerenes and gold nanoparticles.^29,30^ CG models are particularly valuable for dissecting lipid contributions and capturing collective processes such as interfacial lipid reorganization, curvature induction, and local reorganization in hNP–membrane recognition. Moreover, enhanced sampling techniques such as umbrella sampling enable the determination of potential of mean force (PMF) profiles, offering thermodynamic insight into the molecular determinants of the process.^31–33^ The increasing consistency between computational predictions and experimental data highlights the utility of MD and CG models as a rational design tool for engineering selective hNPs.^34^

In this work, we employed CG-MD simulations to systematically investigate how lipid functionalization governs nanoparticle–membrane recognition. Each hNP system consists of a hydrophobic fullerene core (radius ∼1.4 nm) coated by a lipid monolayer. hNPs were designed with lipid coatings based on mammalian-like (N_M_) and tumor-like (N_T_) membrane compositions, and their interactions with both bilayer types were assessed (Figure 1). Unexpectedly, N_M_ did not preferentially associate with mammalian-like membranes (M_M_) but instead exhibited a preferential association with tumor-like membranes (M_T_). To rationalize this counterintuitive result, the hNP composition was deconstructed into its constituent lipid species, and their individual contributions were assessed. Subsequently, based on the role of CHOL in nanoparticle–membrane interactions,^35^ we designed CHOL-enriched hNPs depleted of weakly interacting lipids, which showed marked improvements in membrane affinity and selectivity.

**Figure 1:**
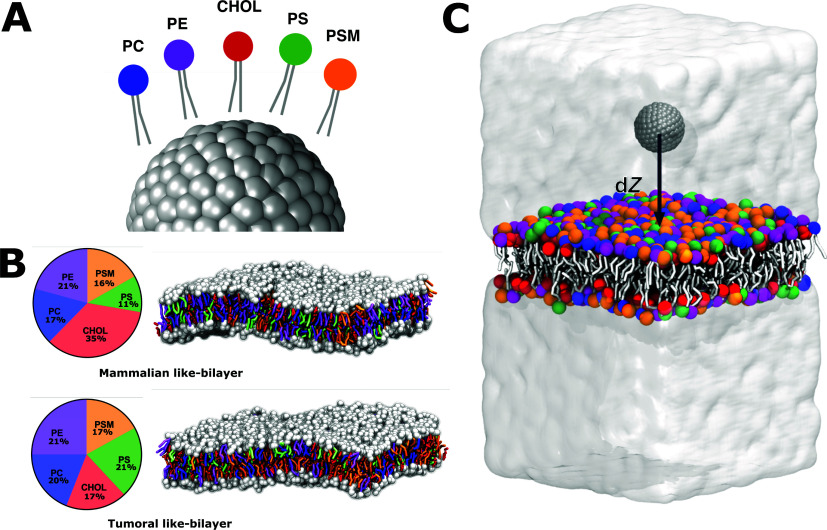
Schematic representation of the systems simulated in this study. (A) Coarse-grained hybrid nanoparticle functionalized with different lipid species: PC (blue), PE (purple), CHOL (red), PS (green), and PSM (orange), illustrating the lipid corona used throughout the study. The hNP consists of a hydrophobic fullerene core (radius ∼1.4 nm) coated by a lipid monolayer, with hydrophobic tails oriented inward and polar headgroups facing the aqueous environment. (B) Lipid compositions and representative snapshots of the M_M_ and M_T_, composed of the same lipid species arranged in distinct relative proportions (see Table S1). (C) Simulation setup showing the interaction between the lipid-functionalized nanoparticle and the complex lipid bilayer. The reaction coordinate d*Z* is defined as the center-of-mass distance between the hNPs and the bilayer.

Together, our results provide mechanistic evidence that hNP lipid composition, rather than lipidome mimicry, governs nanoparticle–membrane interactions, challenging the assumption that mimicry alone is sufficient for targeting. These findings highlight rational lipid design as a key strategy for engineering hNPs with enhanced preference for tumor-like membranes.

Initially, we calculated free-energy profiles to evaluate whether lipid-mimicking hNPs provide a thermodynamic advantage for recognition and insertion. For this, M_M_ and M_T_, along with their corresponding lipid-matched hNPs (N_M_ and N_T_), were utilized. For M_M_, both N_M_–M_M_ and N_T_–M_M_ profiles remain predominantly repulsive, with no evidence of stable binding. Accordingly, both profiles were repulsive across the entire reaction coordinate, exhibiting maxima of approximately +60 and +65 kcal·mol^–1^, respectively, and no detectable binding minima (Figure 2B). Electrostatic interactions became relevant at ∼4.5 nm, consistent with the 1.1 nm Coulomb cutoff of the Martini 2 force field,^36^ yet they remained unfavorable as the hNP approached the bilayer. In contrast, M_T_ exhibited favorable free-energy minima, indicating enhanced affinity toward the softer, CHOL-depleted membrane. Specifically, N_M_ displayed a broad stabilizing minimum spanning ∼1–6 nm, whereas N_T_ showed a narrower well between ∼5 and 3 nm (Figure 2C). At distances of 6–7 nm, hNPs remained outside the interaction range (Figure 2A). Consistent with these free-energy trends, interfacial lipid reorganization profiles further differentiate M_M_ and M_T_ systems. Figure 2E reports directional contact events between M_M_ and N_M_ (M_M_ → N_M_ and N_M_ → M_M_), whereas Figure 2F shows the corresponding contact events between M_M_ and N_T_ (M_M_ → N_T_ and N_T_ → M_M_). In tumor-like systems, panel H describes lipid transfer between M_T_ and N_M_ (M_T_ → N_M_ and N_M_ → M_T_), while panel I reports directional persistent contact events between M_T_ and N_T_ (M_T_ → N_T_ and N_T_ → M_T_). Collectively, these results indicate that M_T_ can support transitions to stable binding states and enhanced interfacial lipid reorganization, particularly for N_M_, in agreement with the reported role of lipid composition in modulating hNP affinity.^37^

**Figure 2:**
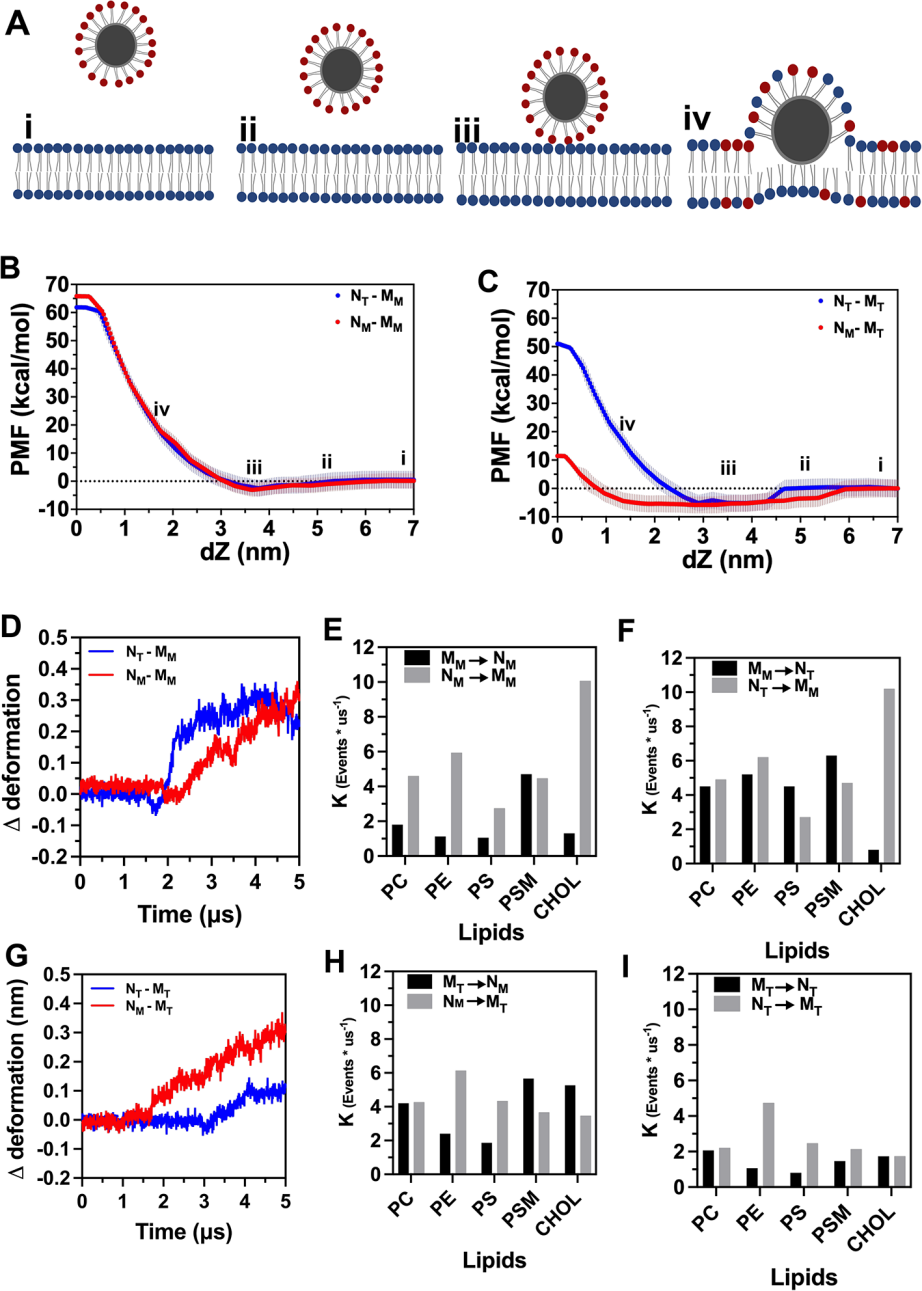
Interaction of hNPs with composition-matched bilayers (M_M_and M_T_). (A) Schematic representation of the main stages of nanoparticle–membrane interaction: (i) approach, (ii) onset of long-range interactions, (iii) interfacial contact, (iv) interfacial association. (B, C) Potentials of mean force (PMFs) describing the free-energy landscape of interaction with M_M_ and M_T_ bilayers. (D, G) Time evolution of membrane deformation during unbiased MD simulations of hNP adsorption. (E, F, H, I) Average rates of persistent lipid contact events (*k*), calculated as the mean number of persistent events across replicas divided by the total simulation time, indicating more pronounced interfacial lipid reorganization in M_T_ than M_M_.

In addition to describing the free-energy landscape of hNP–membrane interactions, unbiased simulations were performed to evaluate the interfacial behavior of N_M_ and N_T_ in M_M_ and M_T_. Consistent with the PMF profiles, the trajectories show sustained interaction with the membrane interface, but not stable insertion into the bilayer interior for the mixed-composition nanoparticles. PMF convergence analyses are provided in the Supporting Information (see Figures S1–S8). Among the four systems, N_M_–M_T_ displayed the most favorable interaction pattern, remaining closer to the membrane and reaching a deeper interfacial position than the others, yet still without penetrating into the bilayer center. By contrast, N_T_ remained predominantly surface-associated (Figure S9). This contrasts with previous Martini studies of lipid NPs, which reported surface anchoring as the dominant outcome.^38^ Differences in the magnitude and temporal evolution of local membrane deformation, defined as the bilayer surface displacement relative to the initial undeformed state, between M_M_ and M_T_ during hNP association with the membrane were captured in the deformation profiles (Figure 2D and 2G). In M_M_, N_T_ induced larger and earlier deformations than N_M_, indicating a stronger perturbation of the more rigid, CHOL-rich membrane. In contrast, in M_T_ the trend was reversed: N_M_ produced the strongest and most progressive deformation, whereas N_T_–M_T_ interactions resulted in only shallow surface distortions. These cross-interaction patterns indicate that maximal membrane deformation arises from lipid and mechanical complementarity rather than composition matching. We note that all simulations used planar bilayers under fully periodic boundary conditions, which suppress curvature relaxation in response to the leaflet stress asymmetry induced by hNP insertion. As discussed by Foley and Deserno,^39^ this constraint is expected to lead to an underestimation of insertion-driven membrane deformation magnitudes. Future work implementing semiperiodic boundary conditions in the Martini framework would allow explicit quantification of this effect. The comparative deformation trends across systems are nonetheless considered robust, as they reflect underlying thermodynamic and compositional differences between systems that are unlikely to be qualitatively reversed by curvature relaxation.^39^ Lipid redistribution analysis (Figure S9) further emphasized this asymmetry: N_M_ lost approximately 33% of its initial CHOL coating, whereas N_T_ retained most of its original composition, losing only ∼12% of CHOL. Around 80% of all persistent events were directed from the bilayer to the nanoparticle, reinforcing that N_M_ promotes stronger interfacial remodeling, while N_T_ remains mostly surface-bound (Figure S10). The persistent contact-event rate (*k*) values (Figure 2E–I) revealed that CHOL plays a pivotal role in modulating interfacial lipid reorganization and affinity. Because *k* was not normalized by lipid abundance, these profiles reflect absolute, composition-dependent contributions rather than per-lipid probabilities. Although compositional mismatch between the nanoparticle corona and the membrane may provide a favorable entropic contribution to interfacial lipid reorganization, the observed *k* values should not be interpreted as purely thermodynamic quantities, since they also depend on lipid mobility and diffusive access to the nanoparticle surface. In addition, explicit entropy/enthalpy decomposition is not straightforward within the Martini framework, because coarse-graining alters the underlying entropy–enthalpy balance.^40,41^ Leaflet-resolved analysis further showed that, upon membrane association, CHOL initially bound to the hNP is rapidly released and redistributed between both membrane leaflets (see Figure S11), supporting a dynamic role of CHOL in interfacial lipid reorganization rather than a simple one-way transfer process. In M_M_, CHOL acts as a structural stabilizer that reduces interfacial reorganization dynamics and resists penetration, while its depletion in M_T_ enhances local flexibility, promoting deformation and lipid flux. Remarkably, nanoparticle affinity was higher when the dominant persistent contact events originated from the nanoparticle toward the membrane, as this direction reflects a coordinated adaptation of both surfaces, leading to more stable interfacial coupling. Conversely, when the dominant persistent contact events occurred from the membrane to the nanoparticle, the process was less coordinated and induced weaker structural rearrangements. Together, these findings demonstrate that CHOL is the primary regulator of the balance between rigidity and fluidity at the nano–bio interface, governing both the magnitude and the directionality of interfacial lipid reorganization that define the stability and affinity of nanoparticle–membrane interactions.

To determine the role of CHOL as a key component in recognition and its influence on membrane deformation and lipid reorganization, we calculated free-energy profiles for the insertion of hNPs coated with individual lipids. In M_M_, PMF profiles for PC, PE, PS, and PSM remained favorable and unfavorable across the entire coordinate, reaching +30 to +60 kcal·mol^–1^ at contact distances (d*Z* < 1 nm) (Figure 3A). In contrast, CHOL displayed a pronounced binding well of approximately −200 kcal·mol^–1^, indicating a strong stabilizing role. In M_T_ (Figure 3D), the overall trend was similar but attenuated: PC, PE, PS, and PSM remained positive with lower magnitudes (+5 to +25 kcal·mol^–1^), while CHOL again showed marked affinity with a well of approximately −150 kcal·mol^–1^. These results identify CHOL as the dominant lipid governing hNP-membrane association, consistent with its well-established role in stabilizing nanodomains and modulating membrane mechanics.^42,43^ Persistent contact-event analyses (Figure 3B and 3E) confirmed the lipid-specific asymmetry observed in membrane deformation and *k* profiles (Figure 2). CHOL showed the strongest depletion from the nanoparticle corona, acting as the main modulator of interfacial stability and lipid mobility. Its release was followed by a partial loss of PE, whereas PC, PSM, and PS exhibited limited lipid transfer events, consistent with their preferential location at the outer leaflet and reduced direct contact with the nanoparticle surface. Importantly, interfacial lipid reorganization is initiated earlier and more efficiently in M_T_ than in M_M_, reflecting their enhanced fluidity and faster structural reorganization to accommodate hNP insertion. These results underscore the pivotal role of CHOL as a dynamic regulator of lipid remodeling at the nano–bio interface.^42,43^ Complementarily, Figure S12 quantifies these trends, showing that CHOL and PE exhibited the highest *k* values, confirming their active participation in interfacial reorganization from the bilayer to the nanoparticle. This supports the notion that CHOL-enriched environments favor rapid interfacial lipid reorganization and interfacial adaptation, whereas more ordered or saturated components (i.e., PSM, PC) remain comparatively static, thereby defining the dynamic asymmetry that governs membrane remodeling around hNPs (Figure 3G and 3H).

**Figure 3:**
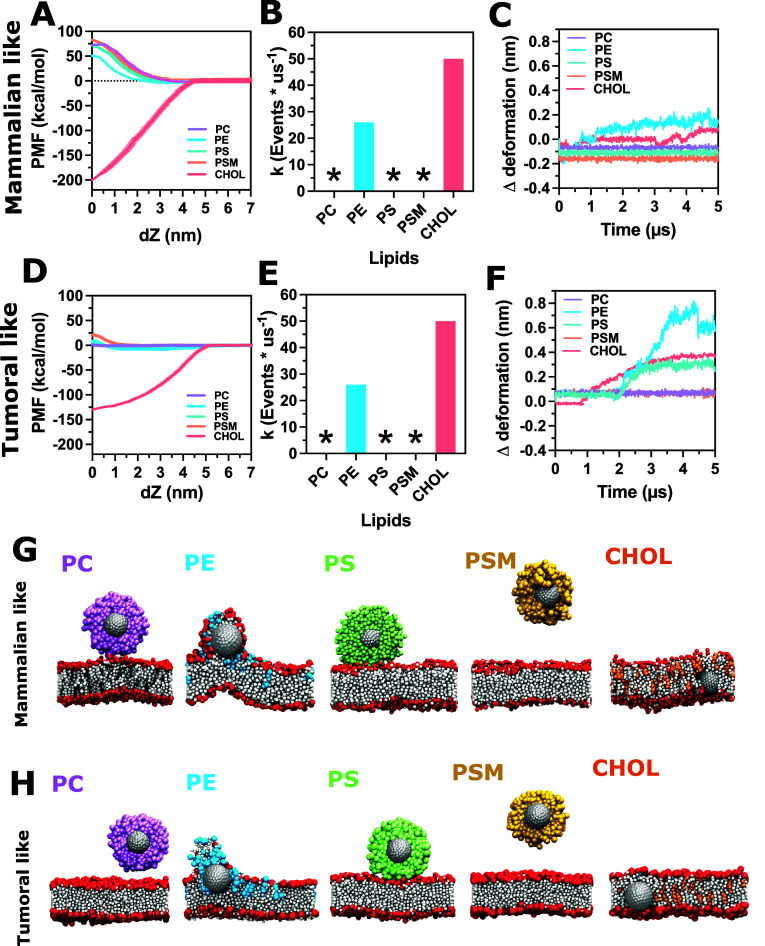
Lipid-specific contributions of hNPs nanoparticles to their interactions with M_M_(A–C, G) and M_T_(D–F, H). (A, D) Potential of mean force (PMF) profiles as a function of the distance from the bilayer midplane (d*Z*) for nanoparticles functionalized with PC, PE, PS, PSM, and CHOL. (B, E) Average rate of persistent contact events (*k*, events·μs^–1^); asterisks (*) indicate time intervals where no *k* values could be determined due to the absence of measurable persistent contact events. (C, F) Temporal variation of the relative membrane deformation induced by each type of hNPs. (G, H) Representative snapshots of equilibrated configurations illustrate the contrasting interaction modes of each lipid coating with M_M_ (G) and M_T_ (H). Each color corresponds to a specific functional lipid (PC, PE, PS, PSM, CHOL).

Additionally, differential lipid positioning within the bilayer and lipid-dependent membrane deformation was evaluated. Figure S13A and S13B show the average position of each lipid type relative to the bilayer midplane (d*Z*). The shaded yellow region (−2 nm ≤ *z* ≤ 2 nm) represents the bilayer interior. CHOL and PSM consistently remained within this region, indicating a tight association with the hydrophobic core and direct participation in maintaining bilayer compactness. In contrast, PC and PE fluctuated farther from the midplane, reflecting their preferential localization at the outer interface. PS occupied an intermediate position—closer to the membrane interior than PC and PE, but without the persistent proximity observed for CHOL or PSM. These positional patterns are consistent with the lipid-specific patterns shown in Figure S12. Deformation profiles (Figure 3C and 3F) revealed that PE produced the strongest perturbations, with values up to 0.8 in M_M_ and 0.3–0.4 in M_T_. CHOL and PSM induced moderate deformation, whereas PC showed negligible effects, consistent with its structural role in bilayers and its lack of specific affinity for external interactions.^35,44^ These results indicate that CHOL and PSM contribute most strongly to membrane proximity and local remodeling, while PC and PE act as unfavorable components: PC stabilizes bilayer architecture but lacks specificity, and PE favors negative curvature and nonlamellar phases, explaining the deformation without stable insertion.^42–44^ PS displayed intermediate behavior, bridging the unfavorable PC/PE group and the favorable CHOL/PSM group. Overall, these findings align with previous reports highlighting CHOL and sphingolipids as key modulators of membrane organization and nanodomain formation.^45–49^ A related mechanistic consideration is the possibility of selective lipid adsorption once the hNP becomes membrane-associated. This effect is expected to be less relevant in the micelle-like aqueous state, but more pronounced at the membrane interface, where local packing constraints and composition-dependent interfacial organization may favor enrichment of specific lipid species near the nanoparticle surface. Core-specific properties, including surface chemistry and core size, may also contribute, although the present simulations were not designed to isolate these effects quantitatively.

Since our initial analyses identified PC and PE as the most unfavorable lipids for hNP–membrane interactions, we systematically evaluated their behavior in binary mixtures with CHOL—a fundamental bilayer organizer and a key component in hNP formulations.^49^ This approach aimed to test whether progressive incorporation of CHOL could compensate for the repulsive character of PC and PE, transforming initially unstable interactions into more favorable ones. PS and PSM were not included, as PSM already displayed partial affinity and PS exhibited intermediate behavior, neither as unfavorable as PC or PE. Therefore, PC/CHOL and PE/CHOL systems provided the most informative models to evaluate CHOL’s compensatory effect under adverse energetic conditions. The 0–100% CHOL gradient was used here as a mechanistic compositional scan to probe CHOL-driven trends and threshold-like behavior, rather than to represent the average CHOL content of physiological plasma membranes. PMF profiles (Figure 4A, 4D, 4G, and 4J) revealed distinct stabilization trends depending on lipid composition. For PC/CHOL mixtures, the free-energy landscape evolved monotonically along the 0–100% CHOL gradient. Pure PC exhibited a highly repulsive profile, but increasing CHOL content progressively reduced the barrier, leading to a deep central minimum at 25PC:75CHOL, consistent with the CHOL condensing effect,^50^ which enhances lipid packing and hydrophobic coupling.^51^ In contrast, PE/CHOL systems exhibited a nonlinear response: pure PE was strongly repulsive, 50PE:50CHOL displayed a metastable minimum corresponding to a surface-adsorbed state, while higher CHOL fractions (75%–100%) reinstated strong repulsion. Notably, the persistence of repulsion for 75PE:25CHOL in the M_T_ indicates that a modest CHOL fraction is insufficient to compensate the unfavorable interfacial contribution of PE when PE remains dominant in the hNP corona, in contrast to PC-rich systems where CHOL more readily promotes interfacial stabilization. This reversal reflects the conical geometry of PE and its tendency toward negative curvature,^52,53^ which CHOL cannot compensate for in PE-rich environments. Furthermore, the persistent contact-event rate (*k*) profiles (Figure 4B, 4E, 4H, and 4K) followed the same compositional trend as the PMFs. In PC/CHOL systems, higher CHOL content led to larger *k* values and reduced fluctuations, indicating more efficient interfacial reorganization and longer nanoparticle residence near the bilayer midplane. Conversely, PE/CHOL mixtures displayed erratic *k* behavior, with transient adsorption and low lipids transfer units efficiency. This nonlinear behavior is consistent with the coupled role of CHOL concentration and phospholipid geometry in regulating lipid packing, as recently proposed by Chen et al.^54^ The membrane deformation profiles (Figure 4C, 4F, 4I, and 4L) confirmed that increasing CHOL content in PC-based nanoparticles enhanced bilayer wrapping and local curvature, whereas PE/CHOL systems produced irregular and short-lived distortions.

**Figure 4:**
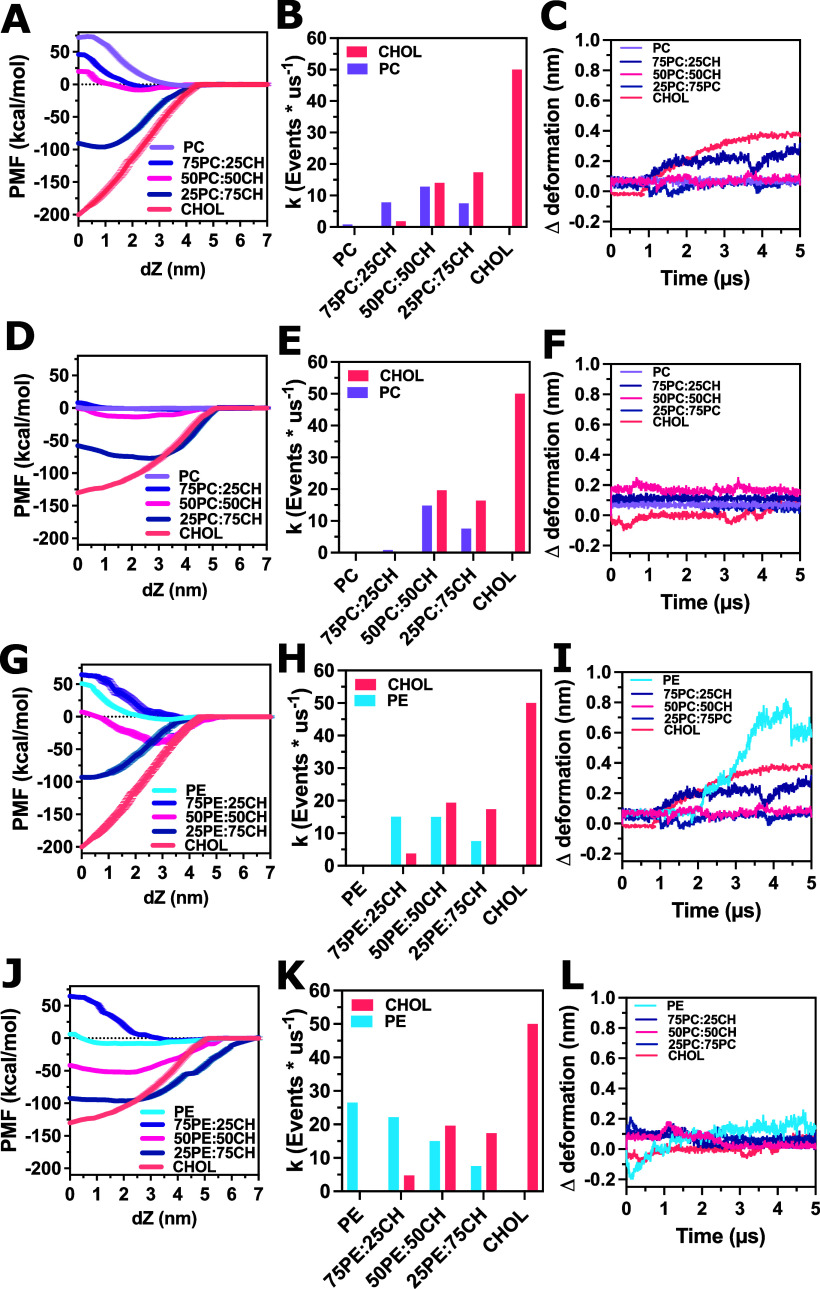
Potential of mean force (PMF) profiles, persistent contact-event rates (*k*), and membrane deformation for hNPs coated with binary mixtures of CHOL and phospholipids across a compositional gradient from 0 to 100% CHOL (25% increments). (A–F) PC/CHOL hNPs in M_M_ (A–C) and M_T_ (D–F). (G–L) PE/CHOL hNPs in M_M_ (G–I) and M_T_ (J–L). The first column (A, D, G, J) shows PMF profiles as a function of the distance along the bilayer normal (d*Z*); the second column (B, E, H, K) shows the average rate of persistent contact events (*k*, events·μs^–1^); and the third column (C, F, I, L) depicts the temporal evolution of local membrane deformation during hNP adsorption.

These trends were consistent with the distance analyses in Figure S14, where the hNP–membrane separation decreased steadily with increasing CHOL content in PC/CHOL systems, confirming that CHOL promotes deeper and more stable insertion. In contrast, PE/CHOL systems showed fluctuating distances, evidencing weaker binding and transient surface association. Complementarily, the membrane-to-nanoparticle lipid transfer rates (Figure S14) revealed that nanoparticles containing higher CHOL fractions induced greater lipid transfer from the membrane, demonstrating that CHOL-rich coronas not only stabilize the interface but also actively recruit surrounding lipids. This behavior underscores CHOL’s role as a bilateral modulator—enhancing both nanoparticle insertion and lipid flow from the membrane to the nanoparticle. Collectively, these results show that CHOL acts as the primary stabilizing factor in hNPs–membrane interactions. In this context, CHOL relocation should be interpreted as an interfacial redistribution process favored upon hNP–membrane contact, rather than as evidence that such high-CHOL membranes are broadly representative of physiological bulk states. In PC/CHOL systems, higher CHOL levels deepen PMF minima, increase *k* values, and promote persistent bilayer deformation and lipid transfer. In contrast, PE/CHOL systems remain unstable, dominated by curvature stress that limits interfacial adaptation. This contrasting behavior aligns with experimental evidence showing CHOL-enriched nanodomains in PC-rich membranes and the destabilizing heterogeneity introduced by PE.^55^ These findings also align with the lipid compositions used in clinically approved lipid nanoparticles, where CHOL and saturated PCs (e.g., DSPC) provide the mechanical stability and fluidity control required for optimal performance.^48,52^ Thus, CHOL not only strengthens the structural integrity of hNPs but also drives active lipid recruitment and interfacial reorganization and mechanisms essential for the rational design of stable, responsive nano–bio assemblies.

Finally, to further dissect the roles of CHOL, PC and PE in hNP selectivity, we generated NM and NT variants in which PC, PE, or both were selectively removed and filled with CHOL (Figure 5). These systems allowed us to evaluate whether the absence of PC and PE, and higher CHOL content, favors insertion and stability of the hNPs. Figure 5 shows that CHOL content strongly modulates the energetic profile of hNP–membrane interactions across both membrane types. In MM, the interfacial PMF values at *z* = 0 (Figure 5A) become progressively more favorable as CHOL increases. Figure 5C illustrates the tendency toward a more inserted and stabilized configuration, but this occurs only when the hNP is completely coated with CHOL. A similar trend is observed in MT (Figure 5B), although with some differences in magnitude, indicating that membrane composition also shapes the energetic response. At the intermediate position (*z* = 2.5 nm), both MM and MT (Figure 5D and 5E) display reduced energetic penalties compared with *z* = 0, suggesting partial stabilization during insertion, consistent with the intermediate configuration represented in Figure 5F. By contrast, at *z* = 5.0 nm, the PMF values in both MM and MT (Figure 5G and 5H) are close to neutral values, indicating that there is no interaction. This is in agreement with Figure 5I, which represents a long distance from the membrane, and where there is no electrostatic interaction. These results support that increasing CHOL content progressively promotes more favorable and stable hNP–membrane interactions, while the presence or absence of specific phospholipids further modulates this effect. Figures S15–S17 expand this analysis by quantifying the energetic consequences of selectively removing PC and PE at *Z* = 0. Figure S16 shows that eliminating PC moderately improves the affinity of N_T_ toward both M_M_ and M_T_, confirming that PC acts as an energetic barrier to insertion. This behavior is consistent with the role of PC as a bilayer-stabilizing lipid that preserves packing order while limiting direct interfacial coupling. Figure S17 further reveals a progressive transition from repulsive to favorable interactions as PC and PE are sequentially removed, with the strongest stabilization observed in the double-depleted systems (NoPC–PE), particularly for N_M_–M_T_ and N_T_–M_T_ systems. These results indicate that PC and PE jointly constitute the principal energetic constraints against deep and stable nanoparticle insertion, and that their removal effectively relieves these limitations.

**Figure 5:**
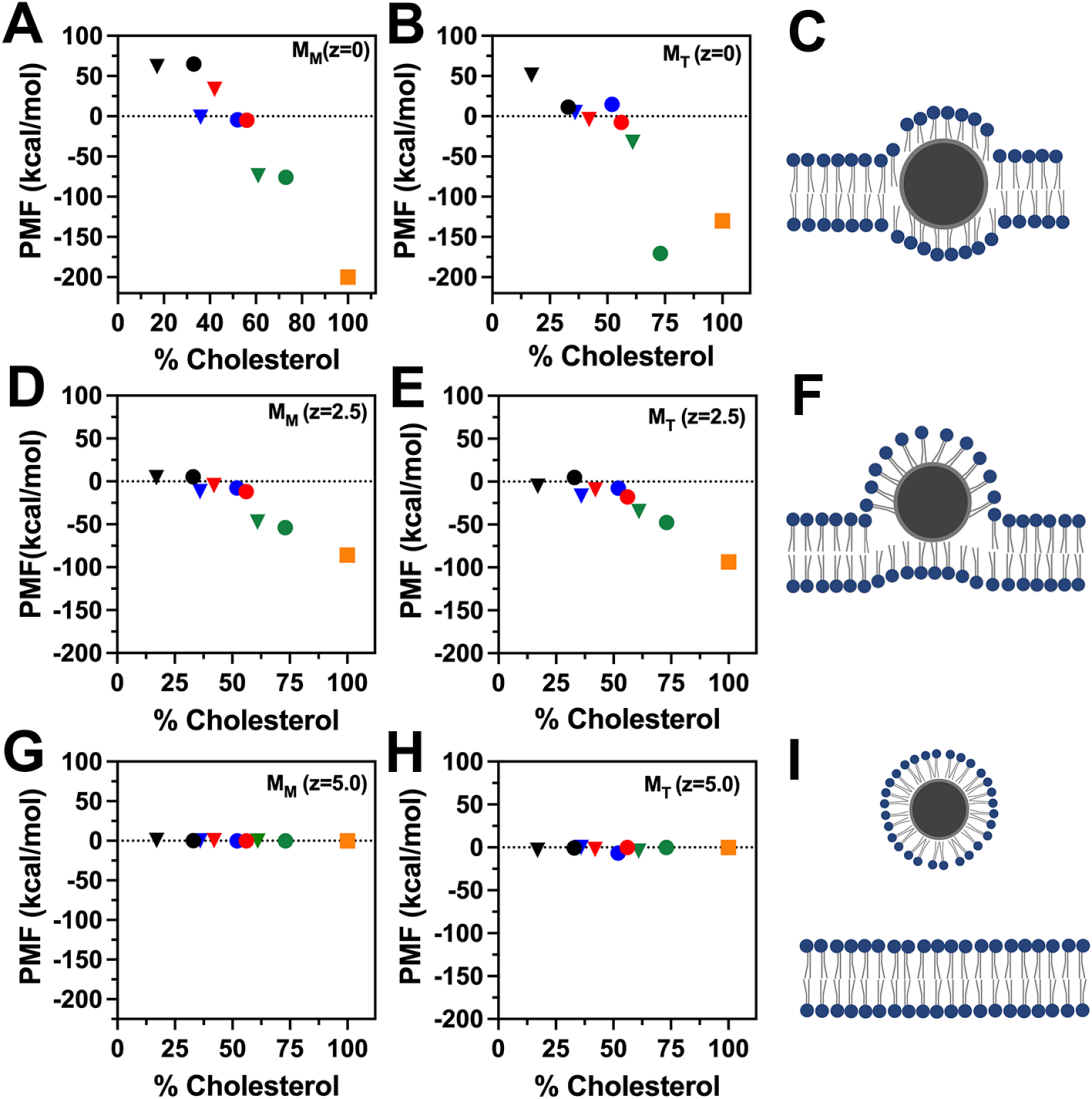
Energetic modulation of nanoparticle–membrane interactions by CHOL content. (A, D, G) PMF values at d*Z* = 0, 2.5, and 5.0 nm for M_M_, and (B, E, H) for M_T_. Circles correspond to N_M_ and triangles to N_T_. Blue, red, and green symbols represent nanoparticles lacking PC, PE, or both PC and PE, respectively, while the orange square denotes the 100% CHOL nanoparticle. (C, F, I) Representative scheme illustrate the progressive insertion and stabilization promoted by increasing CHOL content across both membrane types.

Under these conditions, lipid reorganization becomes dominated by CHOL and PSM, which emerge as the primary energetic mediators of insertion (Figure S15). CHOL, in particular, not only stabilizes the bilayer mechanically but also promotes hydrophobic coupling and the formation of ordered domains that enhance nanoparticle anchoring and retention near the bilayer midplane. This CHOL-driven reorganization accounts for the deep free-energy minima observed in CHOL-rich systems, whereas PE-rich systems remain energetically irregular due to curvature stress and packing frustration. Collectively, Figure 5 and Figures S15–S17 delineate a coherent mechanistic picture in which hNP selectivity arises from the interplay between lipid composition and bilayer structural plasticity. PC and PE primarily act as structural stabilizers that restrict external interactions, whereas CHOL and PSM function as the dominant drivers of affinity, membrane remodeling, and interfacial stability.

From a broader perspective, these findings establish that hNP–membrane recognition is governed by lipid-driven complementarity rather than compositional mimicry. Introducing lipids that energetically penalize interfacial coupling, such as PC or PE, can suppress affinity even when they reflect the target membrane composition. In contrast, enriching nanoparticle coronas with stabilizing and adhesive components, most notably CHOL, enhances insertion, interfacial reorganization, and binding stability. This mechanistic framework directly parallels modern lipid nanoparticle formulations used in biomedical applications, where CHOL is indispensable for stability and function, PC is incorporated only in saturated forms (e.g., DSPC) for mechanical support, and PE is generally avoided due to its destabilizing effects. Together, our results define clear design principles for optimizing hNPs composition and provide a rational basis for engineering selective nanomaterials for therapeutic targeting.^56^

Our findings reveal several overarching principles: (1) CHOL acts as a dominant stabilizer across all systems, enhancing hNP affinity for membranes and promoting ordered bilayer reorganization. By lowering energetic barriers to interaction, CHOL facilitated membrane wrapping and remodeling, reinforcing its central role at the hNP–membrane interface. (2) PE and PC act as energetic barriers, limiting stable hNP–membrane interactions and functioning mainly as structural components rather than active stabilizers. Their removal exposes more favorable interaction landscapes dominated by CHOL and PSM, which enhance adhesion and local ordering. (3) Preferential hNP interaction with M_T_ emerges from the interplay between nanoparticle composition and the bilayer’s capacity for lipid redistribution, local deformation, and interfacial reorganization.

These results show that membrane selectivity is governed by lipid-mediated interfacial organization rather than by compositional mimicry alone. Although PE and PC are abundant in biological membranes, their incorporation into the nanoparticle corona can reduce insertion depth and suppress interfacial reorganization. By contrast, CHOL-enriched coatings promote favorable local packing and bilayer remodeling, enabling stronger and more selective binding.

Together, our results provide a practical design principle for lipid-functionalized hNPs: minimizing the contribution of weakly interacting lipids such as PC and PE, while enriching the corona in CHOL, is sufficient to enhance preferential interaction with M_T_. More broadly, this work highlights rational lipid selection as a strategy to engineer hNPs with improved membrane affinity, selectivity, and delivery potential across biologically relevant interfaces.

## Supplementary Material



## Data Availability

Additionally, the initial structures, simulation input files, and post-processing analysis scripts used in this study are available from the following Zenodo repository: 10.5281/zenodo.18282021.

## Notes

Simulation protocols and computational methods; membrane and nanoparticle compositions; PMF convergence analyses for mixed, single-lipid, binary-mixture, and lipid-depleted hNP systems; unbiased trajectory analyses of nanoparticle–bilayer separation; cumulative persistent contact-event profiles; leaflet-resolved redistribution of nanoparticle-derived cholesterol; lipid-specific persistent contact-event rates; lipid position profiles relative to the bilayer midplane; distance profiles for PC/CHOL and PE/CHOL systems; energetic decompositions of hNP–membrane interactions; structural organization and density profiles of mammalian-like and tumor-like membranes; and supplementary tables with system compositions, simulation conditions, and nanoparticle coverage values (PDF)

## ABBREVIATIONS

N_T_: Tumor-like nanoparticle
N_M_: Mammalian-like nanoparticle
M_M_: Mammalian-like membrane
M_T_: Tumor-like membrane
hNPs: Lipid-functionalized hybrid nanoparticles
PE: Phosphatidylethanolamine
PC: Phosphatidylcholine
CHOL: Cholesterol
PSM: Sphingomyelin
PS: Phosphatidylserine
