# Supplementary material for: Lipid Composition Determines Hybrid Nanoparticle Selectivity: Beyond Membrane Mimicry in Cancer Targeting
Source: Nano Lett. 2026 May 7;26(25):8091–101. doi: 10.1021/acs.nanolett.6c00637 (PMC13330797; doi:10.1021/acs.nanolett.6c00637)
Supplement: Supplementary file 1 [file nl6c00637_si_001.pdf]

Lipid Composition Determines Hybrid Nanoparticle Selectivity: Beyond Membrane Mimicry in Cancer Targeting

L. Gonzalo Espinoza-Arcos,[1,2,5] *Matías Valdés-Peña,*[1] *Juan J. de Pablo,*[3] *Cristian Vilos*[4, 5,6]**,*

Ricardo A. Zamora[7,]**, Riccardo Alessandri,*[2,]** Horacio Poblete*[1,5]**.*

1 Centro de Bioinformática, Simulación y Modelado (CBSM), Facultad de Ingeniería, Campus Talca, Universidad de Talca, Talca, 3465548, Chile.

2 Department of Chemical Engineering, KU Leuven, 3001 Leuven, Belgium.

3 Departments of Chemical and Biological Engineering, Computer Science, and Physics, Tandon School of Engineering, and Courant Institute of Mathematical Sciences, New York University, New York, NY, 11201, United States.

4 Laboratory of Nanomedicine and Targeted Delivery, Faculty of Medicine, Universidad de Talca, Talca, 3465548, Chile.

5 Center for Nanomedicine, Diagnostic and Drug Development (ND3 Center), Universidad de Talca, Talca, 3465548, Chile.

6 Center for Nanoscience and Nanotechnology (CEDENNA), Proyecto CIA250002, Manuel Rodríguez Sur 415, Santiago, Chile.

7 Vicerrectoría Académica, and Escuela de Ingeniería Civil en Bioinformática, Facultad de Ingeniería, Universidad de Talca, Talca, 3465548, Chile.

***Correspondence:** hopoblete@utalca.cl; riccardo.alessandri@kuleuven.be; ricardo.zamora@utalca.cl; cristian.vilos@utalca.cl

Simulation protocol

Coarse-grained MD simulations were performed using GROMACS 2023.4 and the Martini 2 force field.[1,2] All systems were simulated in the NPT ensemble at 310 K and 1 bar. Temperature was controlled using a Berendsen thermostat with τ□ = 1 ps,[3] and pressure with a C-rescale semi-isotropic barostat with τ□ = 4 ps and a compressibility of 3.0×10^{-4} bar^{-1}.[4] Periodic boundary conditions were applied in all directions, and the equations of motion were integrated using a 20 fs time step**.** Nonbonded Lennard–Jones interactions were truncated at 1.1 nm, and electrostatics were treated using the Particle Mesh Ewald (PME) method with a real-space cutoff of 1.1 nm.[5,6]

Membrane Models and Lipids-functionalized Hybrid Nanoparticles

Mammalian-like (M_M) and tumor-like (M_T) bilayers were generated with insane.py under the Martini 2 coarse-grained force field.[7] Both bilayers were modeled as symmetric five-component mixtures (see Fig. S9) (PC, PE, PS, PSM, CHOL), with compositions (mol%) summarized in Table S1. Each bilayer patch measured 20 × 20 nm^2 with an initial box height of 20 nm, followed by energy minimization and equilibrium. Production runs of 10 µs were performed in the NPT ensemble at 310 K and 1 bar with periodic boundary conditions.

Lipid functionalized-hybrid nanoparticles (*hNPs*) were assembled with Packmol by distributing lipids around an F216 fullerene core (216 CNP beads),[8] parametrized according to Monticelli et al.[9] Lipids were oriented with their hydrophobic tails facing the fullerene and their headgroups facing outward, with randomized placement to ensure homogeneous angular distribution. Systems were solvated with water, and counterions (Na^+ and Cl^-) were added to maintain overall electroneutrality. After energy minimization and equilibration, production simulations of 5 µs were carried out under the general simulation settings described above. In all

nanoparticle simulations, the lipid coating remained stably assembled around the fullerene core without any detachment events.

Unbiased Molecular Dynamics Simulations

hNPs were initially positioned ~6.0 nm above the bilayer surface along the membrane normal. After energy minimization and equilibration, during which the hNP coordinates were restrained, the systems were released for production runs of at least 5 µs using a 20 fs time step. To verify computational convergence, three independent replicas were performed for each simulation condition. A full description of the membrane–hNP systems under all conditions is provided in Tables S2–S7. Interfacial lipid reorganization was analyzed from the unbiased MD trajectories as follows.[13] Membrane deformation was quantified as the local displacement of the bilayer surface relative to its initial reference configuration during hNP interaction. Specifically, the membrane surface was monitored over time and compared to the corresponding undeformed bilayer state, allowing us to track the magnitude and temporal evolution of local bilayer distortion induced by the nanoparticle. For each unbiased trajectory, persistent lipid contact events were quantified bidirectionally between nanoparticle-coating lipids and membrane lipids using a 0.6 nm distance cutoff, consistent with the bead–bead contact criterion commonly used in Martini-based analyses. Events were evaluated at the lipid-residue level, and an event was recorded when any bead from a given lipid residue remained within 0.6 nm of the opposite element for longer than 20 ns. Events were classified according to the direction of interaction, i.e., nanoparticle-coating lipids contacting the membrane (NP→Memb) or membrane lipids contacting the nanoparticle surface (Memb→NP). For each lipid class, k was defined as the average rate of persistent contact events across replicas over the total simulation time and is reported in events·μs^{-1}.

$$k_\alpha = \frac{1}{T_{total}}\left(\frac{1}{n_r}\sum_{r=1}^{n_r} N_{\alpha,r}\right)$$

where $N_{\alpha,r}$ is the number of persistent contact events for lipid class α in replica r, n_r is the number of replicas, and T_{total} is the total simulation time. Thus, k_α is reported in events·μs^{-1}.

Calculations of Potentials of Mean Force

To quantify the free energy of hNP–membrane interactions, the potential of mean force (PMF) was computed as a function of the distance between the hNP and the membrane center of mass using the Umbrella Sampling method.[10] The hNP–membrane center of mass distance was divided into 30 windows spaced at 0.2 nm. For each window, the system was simulated for 1 µs with a harmonic restraining potential of 750 kJ·mol^{-1}·nm^{-2} applied between the membrane and the hNP to maintain the target distance.[11] The resulting trajectories were analyzed with the weighted histogram analysis method (WHAM) using the g_wham tool from the GROMACS package, and statistical errors were estimated *via bootstrap resampling* (200 samples).[12-13]

Data availability

Initial structures, simulation input files, and post-processing analysis scripts used in this study are accessible at the following Zenodo repository: https://doi.org/10.5281/zenodo.18282021

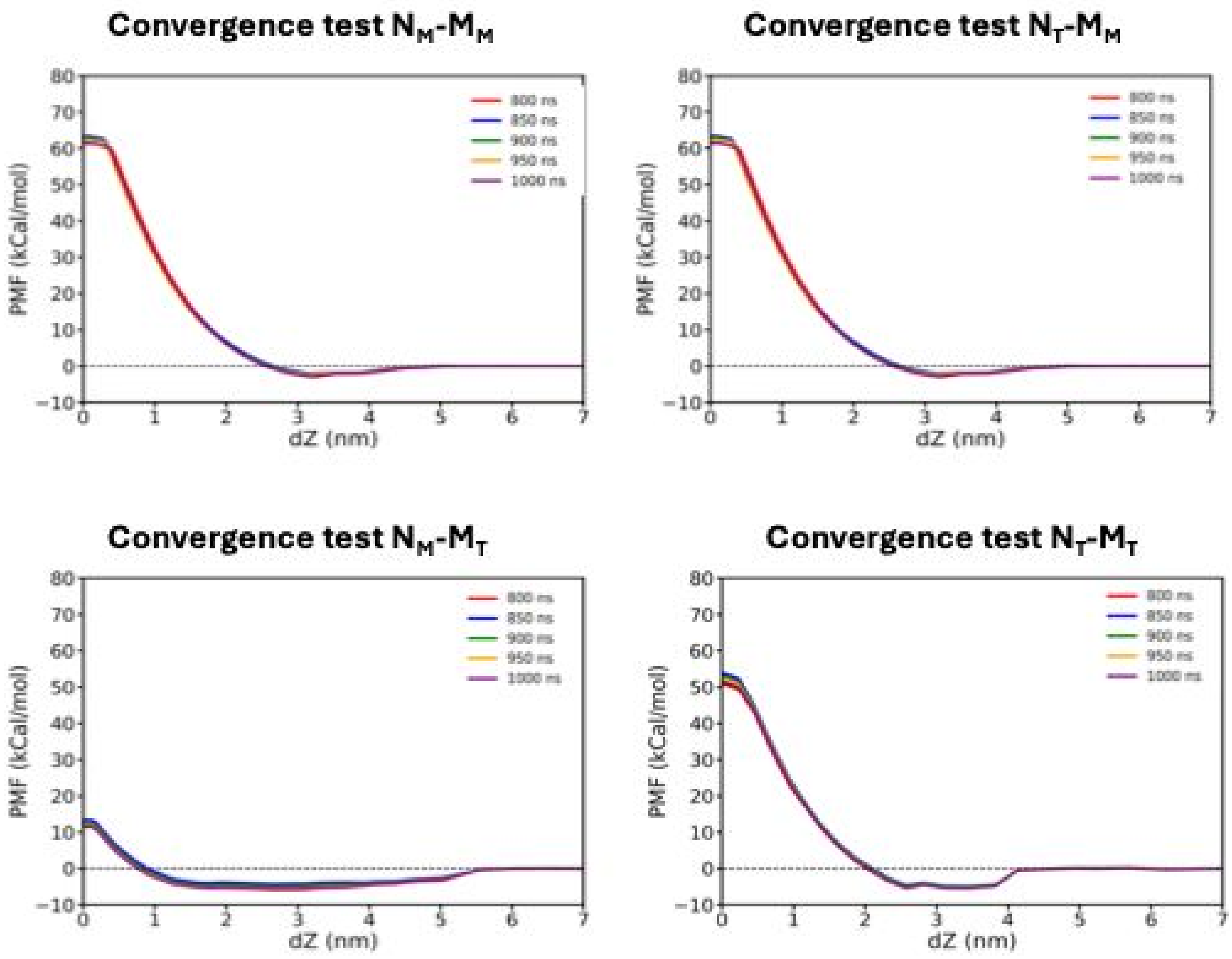

Fig. S1: PMF convergence analysis for mixed-composition hNPs interacting with M_M and M_T.

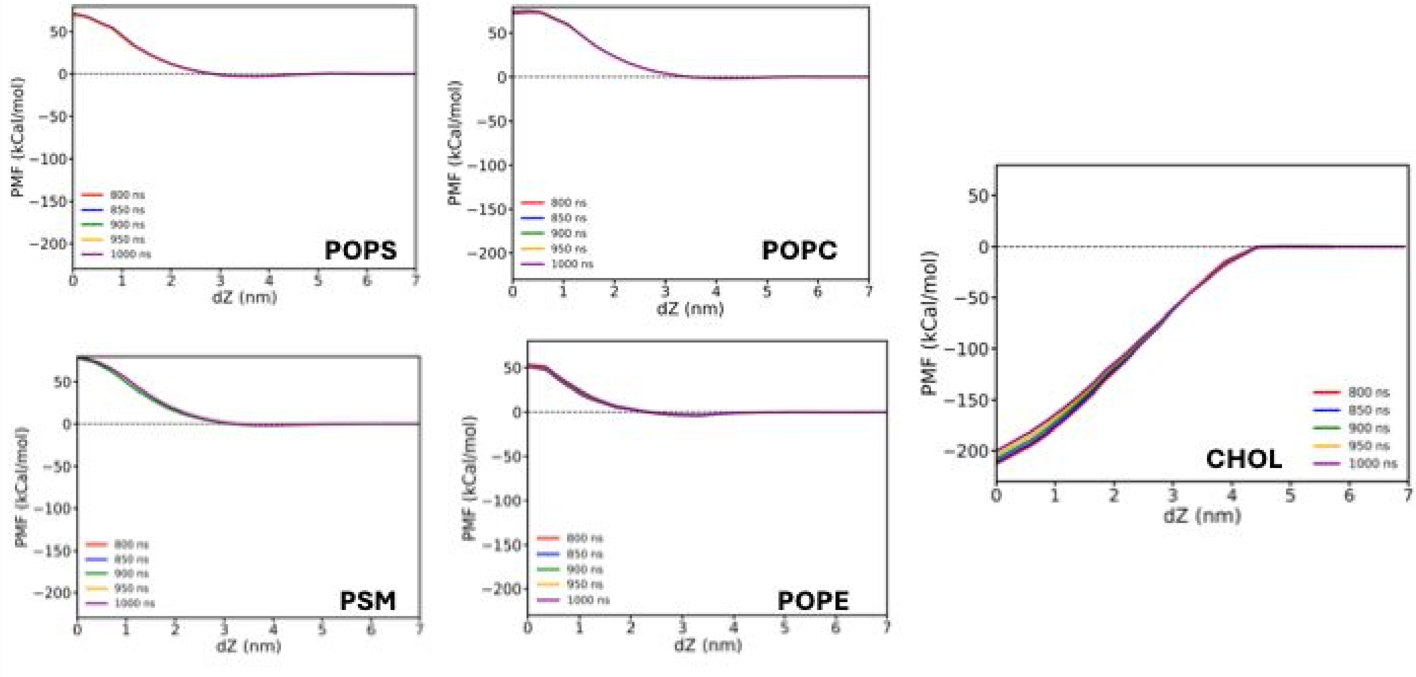

Fig. S2: PMF convergence analysis for single lipids-composition hNPs interacting with M_M.

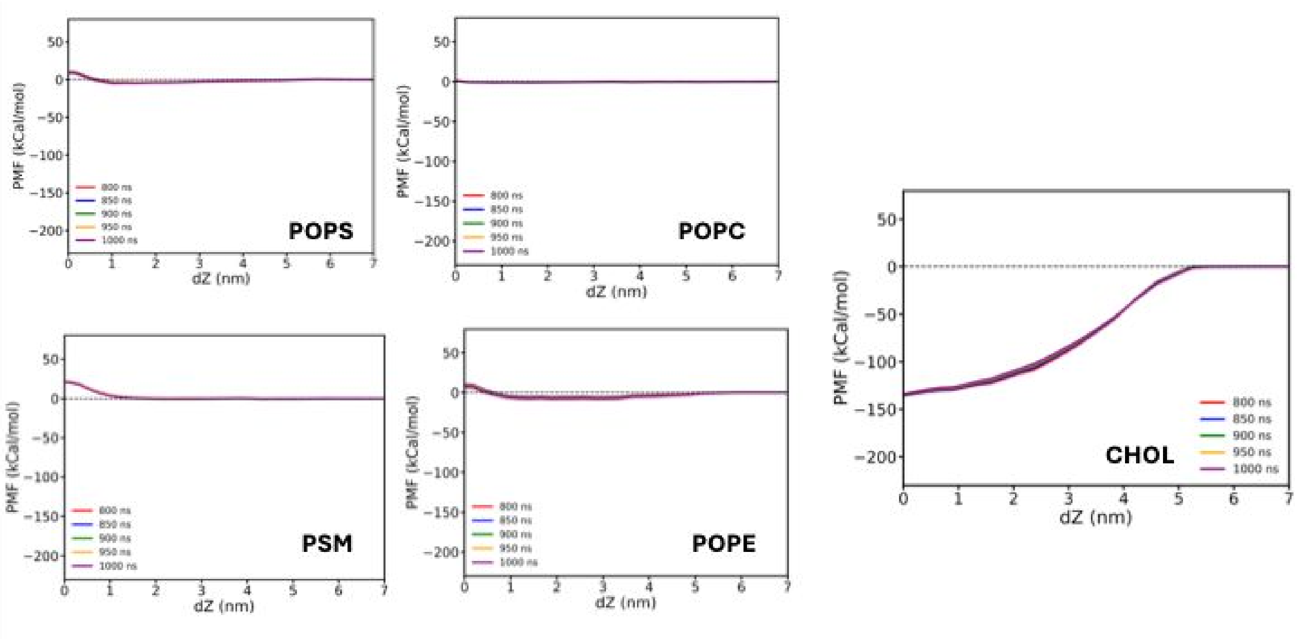

Fig. S3: PMF convergence analysis for single lipids-composition hNPs interacting with M_T.

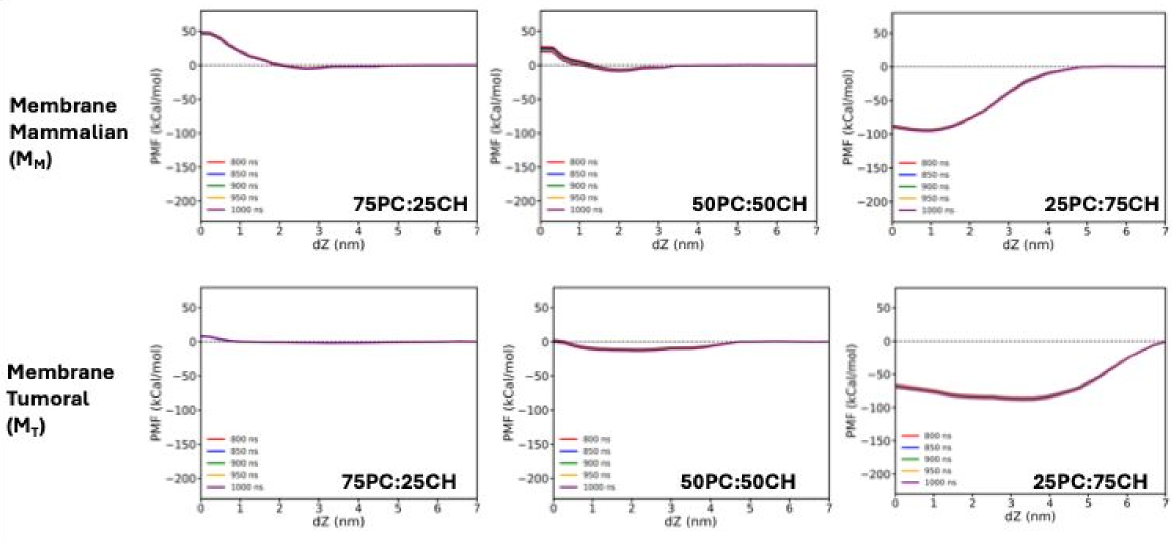

Fig. S4: PMF convergence analysis for PC:CH binaries mixtures lipids-composition hNPs interacting with M_M and M_T.

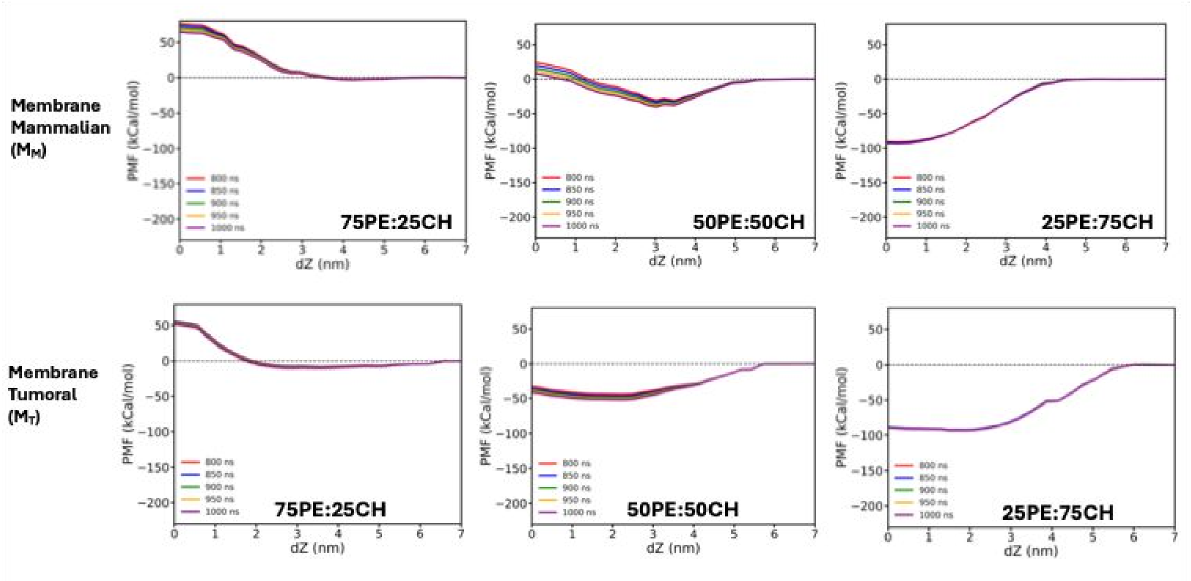

Fig. S5: PMF convergence analysis for binaries mixtures PE:CH slipids-composition hNPs interacting with M_M and M_T.

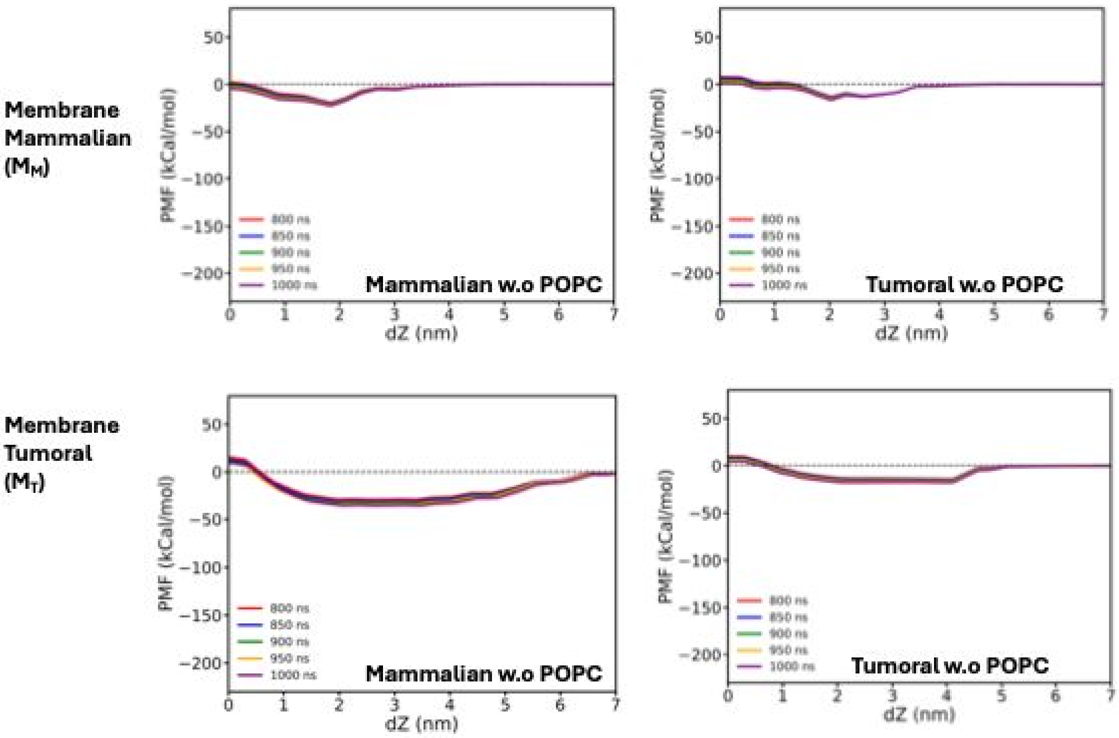

Fig. S6: PMF convergence analysis for binaries mixtures with out POPC (PC) lipids-composition hNPs interacting with M_M and M_T.

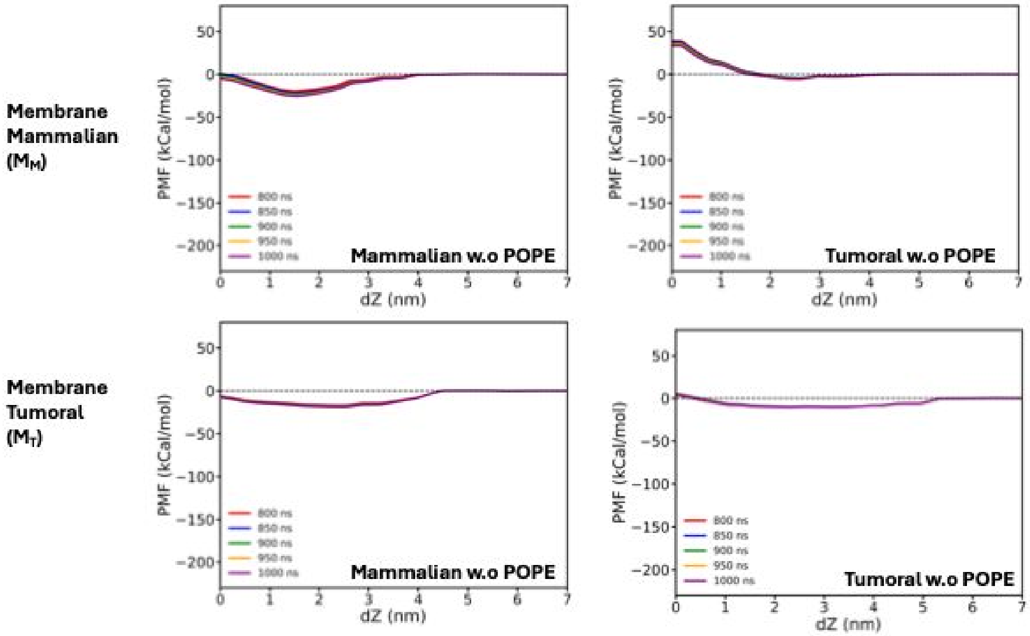

Fig. S7: PMF convergence analysis for binaries mixtures with out POPE (PE) lipids-composition hNPs interacting with M_M and M_T.

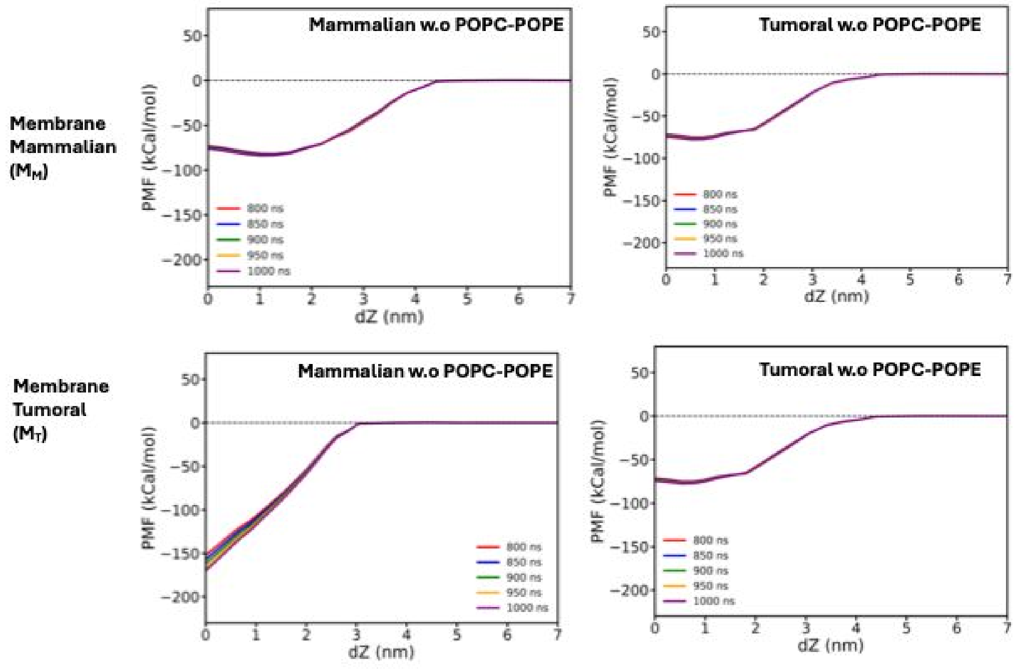

Fig. S8: PMF convergence analysis for binaries mixtures with out POPC (PC) and POPE (PE) lipids-composition hNPs interacting with M_M and M_T.

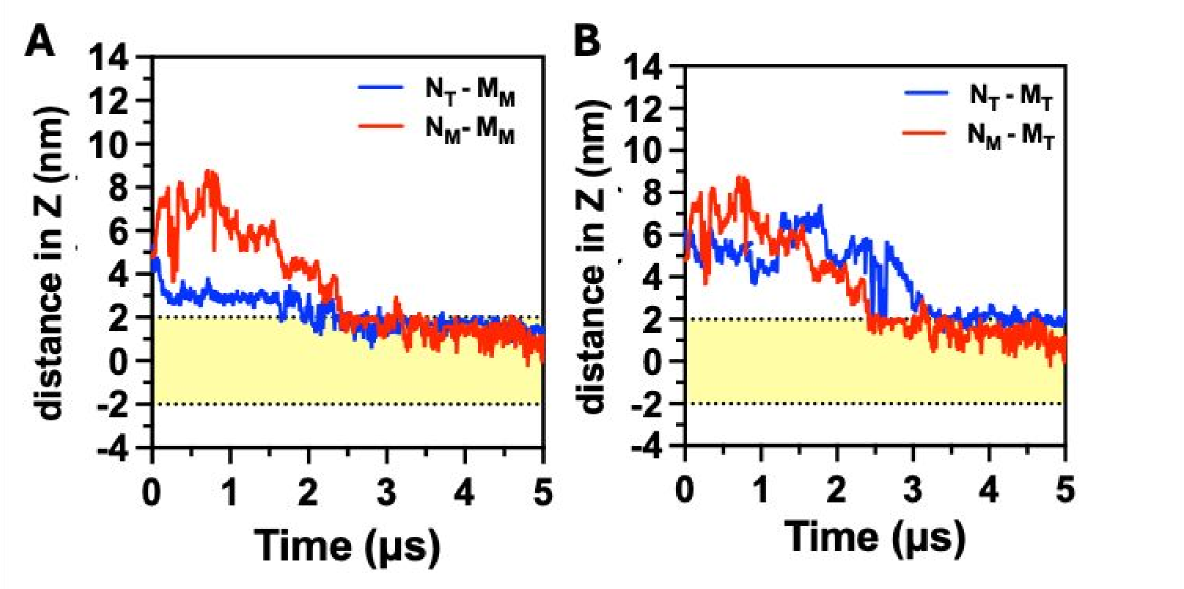

Figure S9. Time evolution of the nanoparticle–bilayer separation (dZ) during unbiased simulations for (A) N_M and (B) N_T interacting with M_M and M_T. The shaded yellow region represents the membrane interior ($-2 \leq z \leq 2$ nm). N_M exhibits closer and more sustained interfacial association both bilayers, particularly in M_T, whereas N_T remains mostly interfacial with larger fluctuations over time.

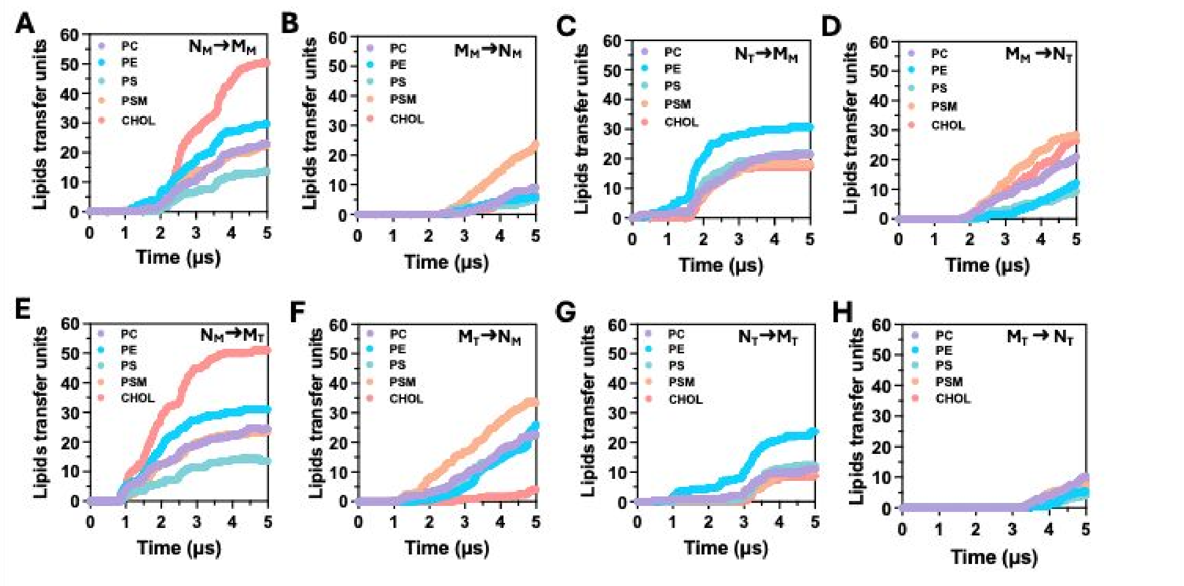

Figure S10. Lipid transfer units between hNPs and membranes. The curves show the cumulative number of lipid persistent contact events (PC, PE, PS, PSM, and cholesterol) over 5 μs of simulation.

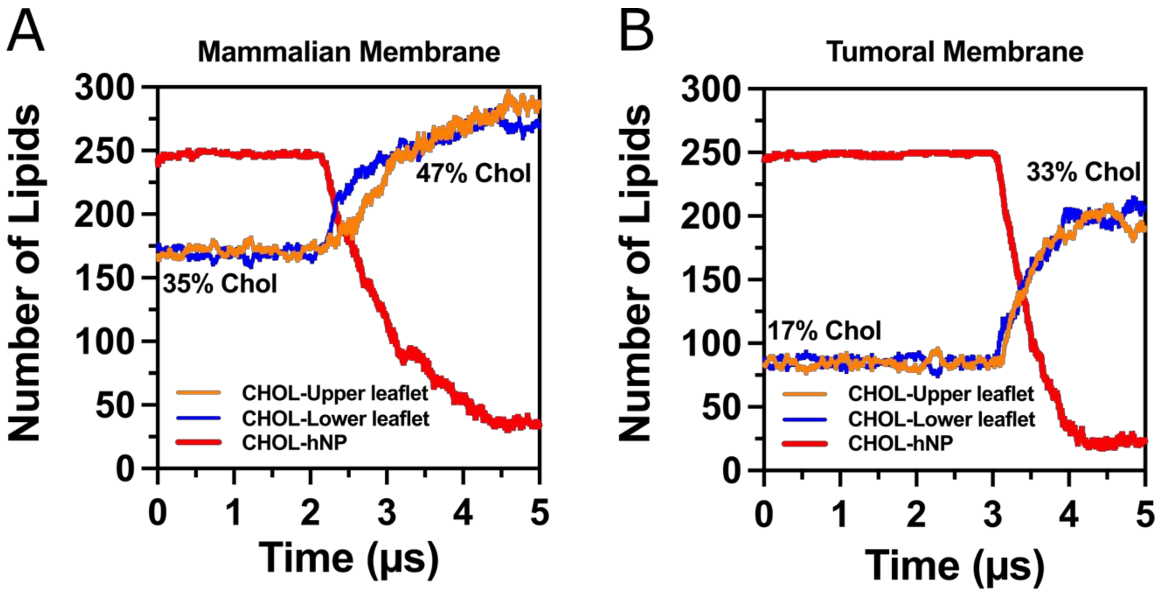

Figure S11. Leaflet-resolved redistribution of nanoparticle-derived cholesterol during hNP–membrane interaction. (A) M_M and (B) M_T. The orange and blue traces represent the number of cholesterol molecules located in the upper and lower membrane leaflets, respectively, whereas the red trace indicates the number of cholesterol molecules that remain associated with the nanoparticle (hNP).

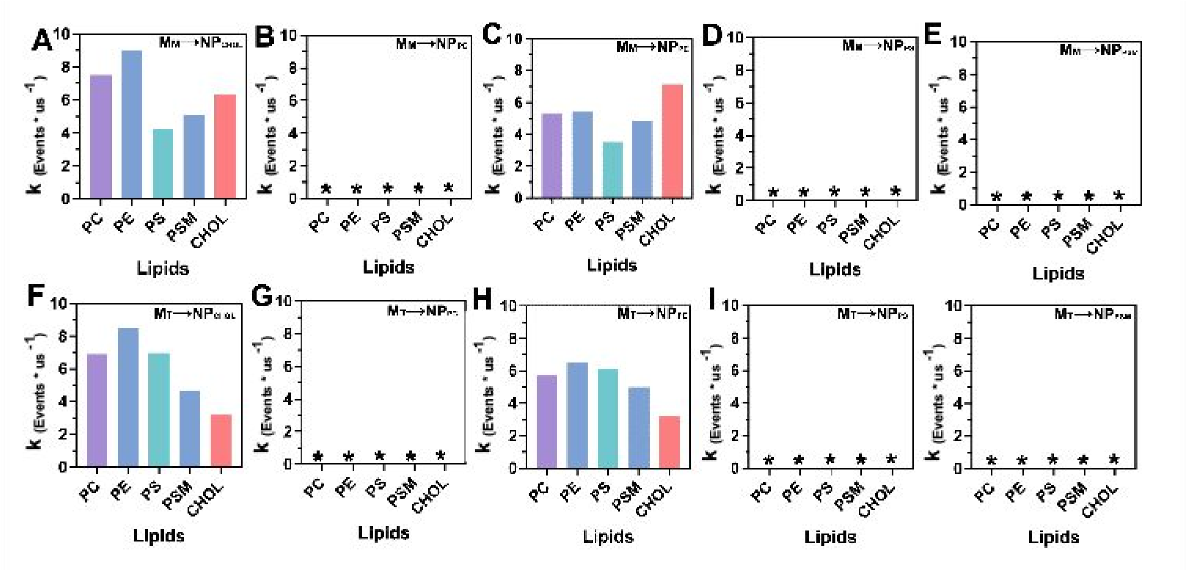

Figure S12. Average rate of persistent contact events (k) from the membrane to the nanoparticle for each lipid species (PC, PE, PS, PSM, and CHOL) in M_M and M_T.

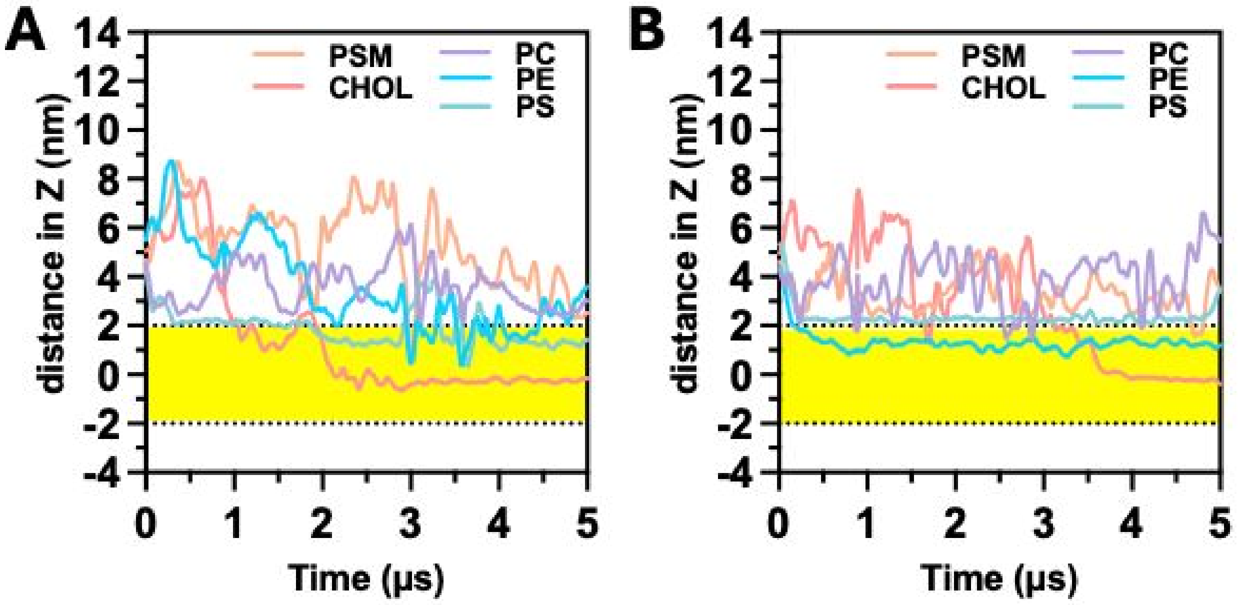

Figure S13. Time-dependent distance profiles (dZ) of PC, PE, PS, PSM, and CHOL relative to the bilayer midplane. The shaded yellow region ($-2 \leq z \leq 2$ nm) denotes the membrane. CHOL and PSM remained consistently close to the bilayer core, whereas PC and PE fluctuated at more external positions. PS maintained an intermediate distance, reflecting partial but transient penetration toward the bilayer interior.

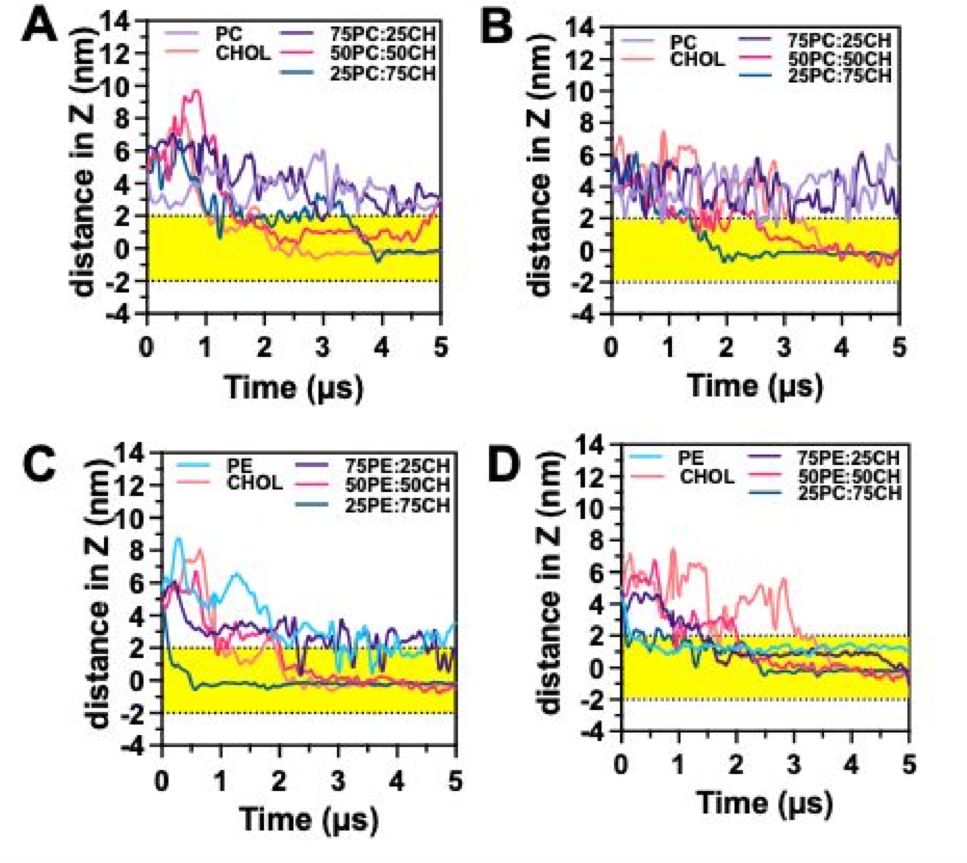

Figure S14. Time evolution of the nanoparticle–bilayer distance distance in Z for PC/CHOL and PE/CHOL systems at increasing cholesterol fractions (0–100%, 25% increments). The yellow band ($-2 \leq z \leq 2$ nm) denotes the bilayer interior. Higher CHOL content in PC-based nanoparticles stabilized insertion near the bilayer midplane, while PE/CHOL systems exhibited fluctuating and transient surface association.

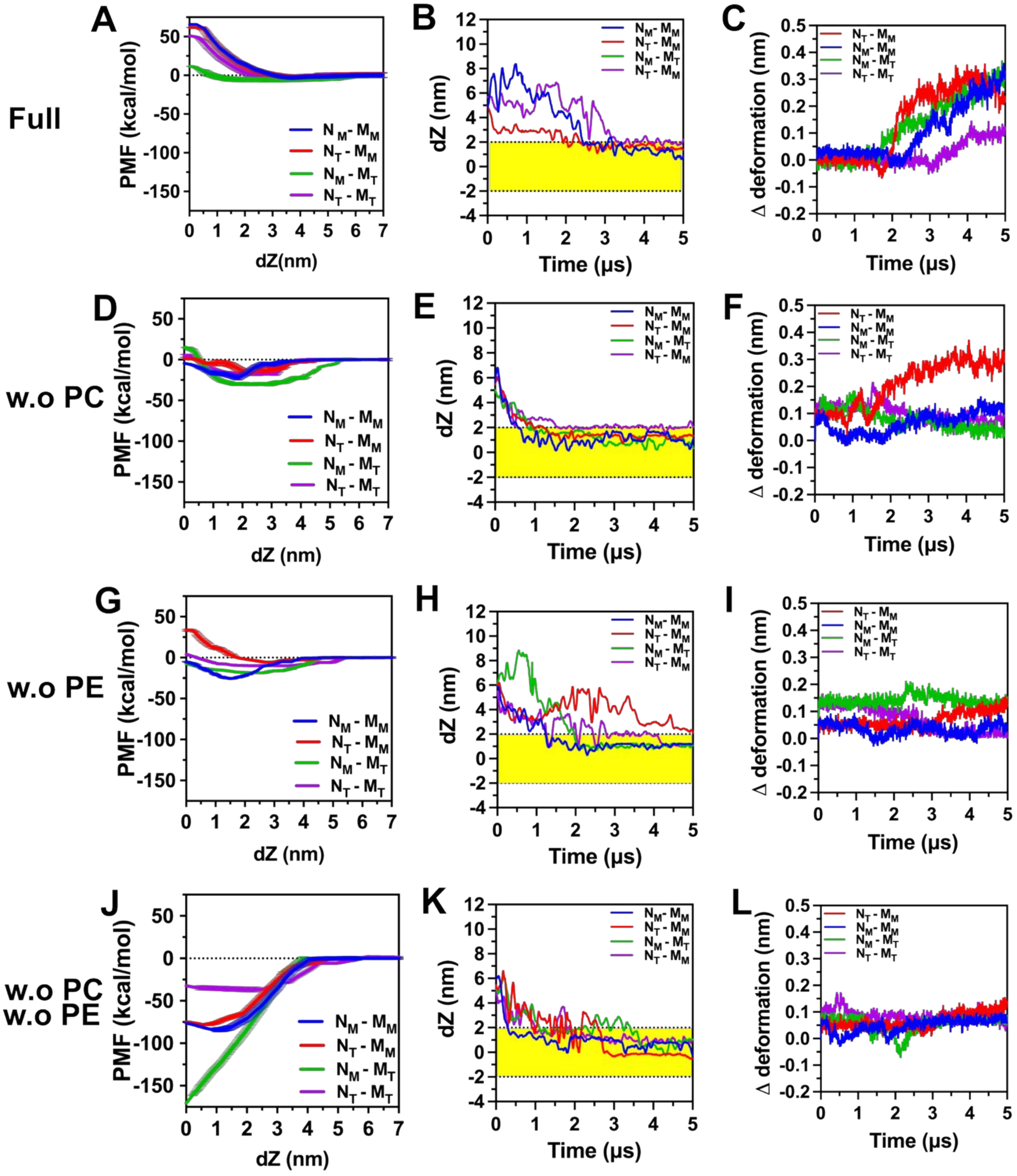

Figure S15. Potential of mean force (PMF) profiles, free dynamics trajectories, and membrane deformation for complete hNPs and their variants with PC, PE, or both species removed. (A–C)

Complete nanoparticles. (D–F) N_M without PC. (G–I) N_M without PE. (J–L) N_M without PC and PE. The first column (A, D, G, J) shows the PMF profiles as a function of the distance along the z-axis (d*Z*). The second column (B, E, H, K) corresponds to the time evolution of the nanoparticle–membrane center-of-mass distance over 5 µs of simulation. The third column (C, F, I, L) presents the relative bilayer deformation profiles. The yellow region indicates the position of the membrane ($-2 \leq z \leq 2$ nm)

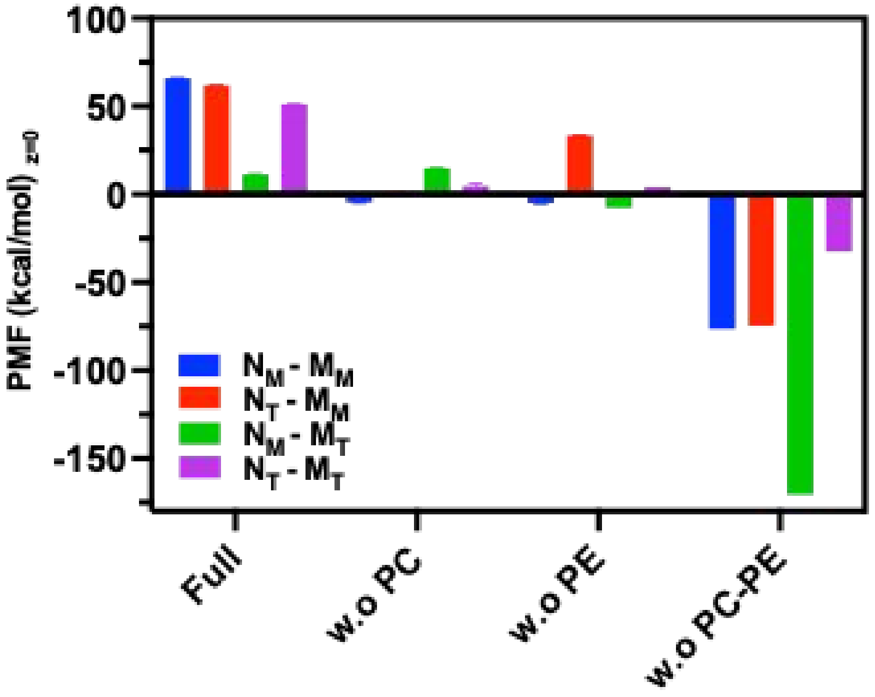

Figure S16. Energetic decomposition of hNPs–membrane interactions at Z = 0. (A) Complete systems showing N_M and N_T interacting with M_M and M_T. (B) Systems lacking phosphatidylcholine (NoPC). (C) Systems lacking phosphatidylethanolamine (NoPE). (D) Systems lacking both PC and PE (NoPC–PE). Bars represent the relative energetic contributions (in kcal·mol^{-1}) for each nanoparticle–membrane combination, as in Figure 5. Blue, red, green, and magenta correspond to N_M–M_M, N_T–M_M, N_M–M_T, and N_T–M_T systems, respectively.

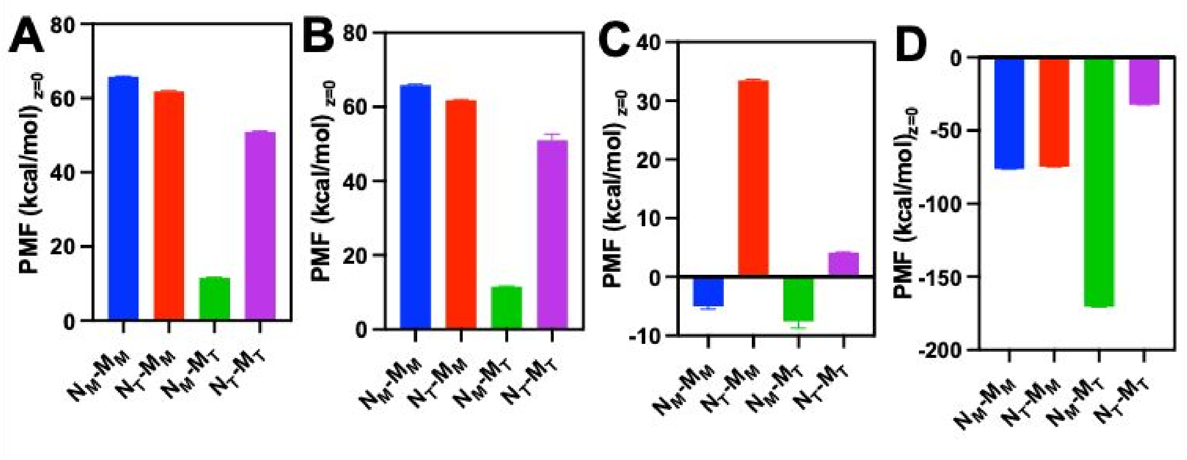

Figure S17. Energetic decomposition of hNPs–membrane interactions at Z = 0. (A) Complete systems showing N_M and N_T interacting with M_M and M_T. (B) Systems lacking phosphatidylcholine (NoPC). (C) Systems lacking phosphatidylethanolamine (NoPE). (D) Systems lacking both PC and PE (NoPC–PE). Bars represent the relative energetic contributions (in kcal·mol^{-1}) for each nanoparticle–membrane combination, as in Figure 5. Blue, red, green, and magenta correspond to N_M–M_M, N_T–M_M, N_M–M_T, and N_T–M_T systems, respectively.

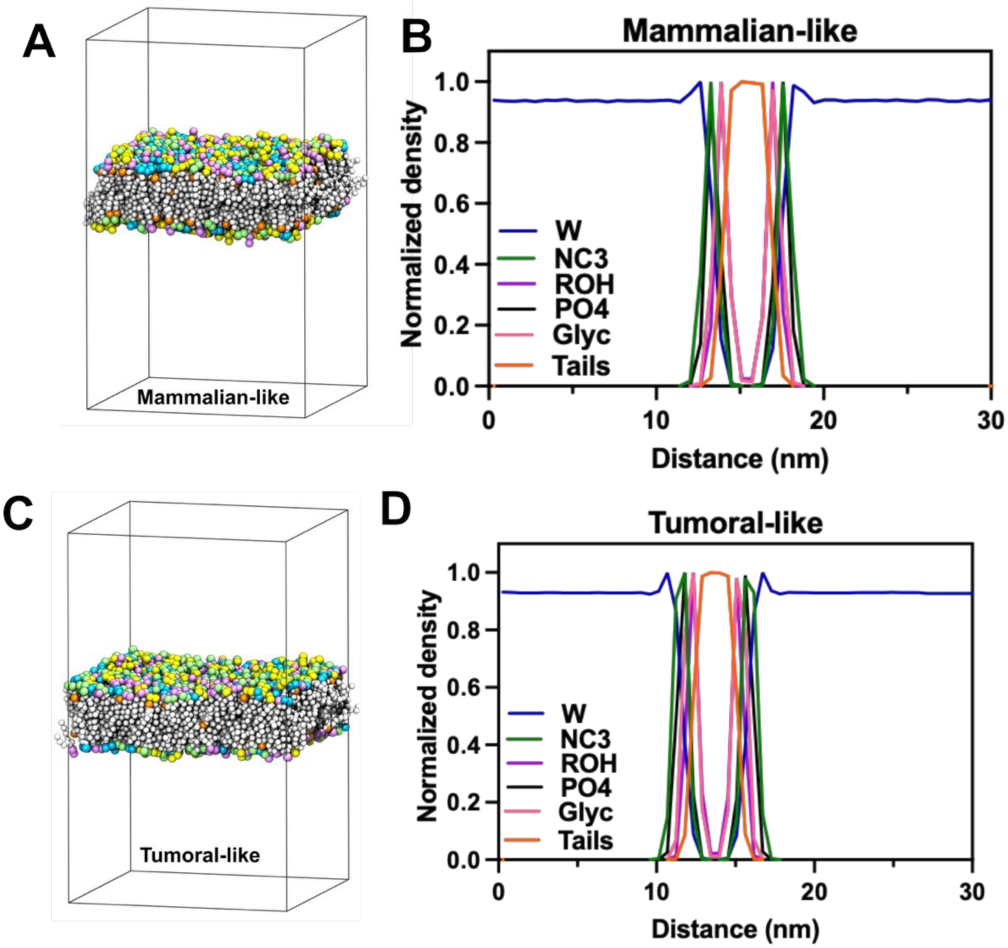

Figure S18: Structural organization of M_M and M_T. (A, C) Representative snapshots of M_M and M_T, respectively. (B, D) Normalized density profiles of water and lipid components along the bilayer normal, highlighting compositional differences between M_M and M_T.

Table S1. Percentage and lipid composition of N_M and N_T

**Membrane Type**	PC%	PE%	PS%	PSM%	CHOL%	Total
Mammalian-like	17	21	11	16	35	100
Tumoral-like	20	25	21	17	17	100

Table S2. Complex lipids hNPs compositions

**Nanoparticle Type**	PC	PE	PS	PSM	CHOL	Total	Av. Covering (%)
Mammalian-like	26	31	17	24	51	149	97.38 ± 1.06
Tumoral-like	30	38	32	25	26	151	98.78 ± 0.47

Table S3. Single lipids hNPs

**Nanoparticle Type**	Lipid	RG (nm)	N° Waters	N° ions	Simulated time (μs)	Independent runs	Average covering (%)
CHOL	150	1.44	24565	0	5	3	45.27 ± 6.3
CHOL	250	1.458	24565	0	5	3	99.1 ± 0.3
POPC	150	1.535	26380	0	5	3	99.5 ± 0.2
POSM	150	1.516	26735	0	5	3	98.7 ± 0.3
POPE	150	1.530	26829	0	5	3	99.2 ± 0.5
POPS	150	1.510	24392	150	5	3	98.6 ± 0.3

Table S4. System informations for hNPS-membranes

**Nanoparticle Type**	Membrane	Simulated time (μs)	Independent runs
CHOL	Mammalian-like	5	3
	Tumoral-like	5	3
POPC	Mammalian-like	5	3
	Tumoral-like	5	3
POSM	Mammalian-like	5	3
	Tumoral-like	5	3
POPE	Mammalian-like	5	3
	Tumoral-like	5	3
POPS	Mammalian-like	5	3
	Tumoral-like	5	3

Table S5 PC:CHOL Mixed nanoparticles

**Nanoparticle Type**	Membrane	Simulated time (μs)	Independent runs	Average Covering (%)
Mixture 75PC:25CHOL	Mammalian-like<br>Tumoral-like	5<br>5	3<br>3	98.78 ± 0.47
Mixture 50PC:50CHOL	Mammalian-like<br>Tumoral-like	5<br>5	3<br>3	97.68 ± 1.02
Mixture 25PC:75CHOL	Mammalian-like<br>Tumoral-like	5<br>5	3<br>3	97.08 ± 1.47

Table S6. PE:CHOL Mixed Nanoparticles

**Nanoparticle Type**	Membrane	Simulated time (μs)	Independent runs	Average Covering (%)
Mixture<br>75PE:25CHOL	Mammalian-like<br>Tumoral-like	5<br>5	3<br>3 (0)	99.13 ± 0.21
Mixture<br>50PE:50CHOL	Mammalian-like<br>Tumoral-like	5<br>5	3<br>3	98.8 ± 0.5
Mixture<br>25PE:75CHOL	Mammalian-like<br>Tumoral-like	5<br>5	3<br>3	97.68 ± 0.9

Table S7. Nanoparticles with specific lipids (PC-PE)

**Nanoparticle Type**	Membrane	Simulated time (μs)	Independent runs	Average Covering (%)
Mammalian NoPE	Mammalian-like<br>Tumoral-like	5<br>5	3<br>3	97.6 ± 2.1
Tumoral NoPE	Mammalian-like<br>Tumoral-like	5<br>5	3<br>3	96.8 ± 1.8
Mammalian-like NoPC	Mammalian-like<br>Tumoral-like	5<br>5	3<br>3	98.1 ± 0.9
Tumoral NoPC	Mammalian-like<br>Tumoral-like	5<br>5	3<br>3	98.8 ± 0.5
Mammalian-like No PEPC	Mammalian-like<br>Tumoral-like	5<br>5	3<br>3	96.4 ± 1.3
Tumoral No PEPC	Mammalian-like<br>Tumoral-like	5<br>5	3<br>3	97.01 ± 1.4

REFERENCES

(1) Marrink, S. J.; Risselada, H. J.; Yefimov, S.; Tieleman, D. P.; de Vries, A. H. The MARTINI Force Field: Coarse Grained Model for Biomolecular Simulations. *J. Phys. Chem. B* **2007**, *111* (27), 7812–7824. https://doi.org/10.1021/jp071097f.

(2) Abraham, M. J.; Murtola, T.; Schulz, R.; Páll, S.; Smith, J. C.; Hess, B.; Lindahl, E. GROMACS: High Performance Molecular Simulations through Multi-Level Parallelism from Laptops to Supercomputers. *SoftwareX* **2015**, *1–2*, 19–25. https://doi.org/10.1016/j.softx.2015.06.001.

(3) Berendsen, H. J. C.; Postma, J. P. M.; van Gunsteren, W. F.; DiNola, A.; Haak, J. R. Molecular Dynamics with Coupling to an External Bath. *J. Chem. Phys.* **1984**, *81* (8), 3684–3690. https://doi.org/10.1063/1.448118.

(4) Bussi, G.; Donadio, D.; Parrinello, M. Canonical Sampling through Velocity Rescaling. *J. Chem. Phys.* **2007**, *126* (1), 014101. https://doi.org/10.1063/1.2408420.

(5) Essmann, U.; Perera, L.; Berkowitz, M. L.; Darden, T.; Lee, H.; Pedersen, L. G. A Smooth Particle Mesh Ewald Method. *J. Chem. Phys.* **1995**, *103* (19), 8577–8593. https://doi.org/10.1063/1.470117.

(6) Darden, T.; York, D.; Pedersen, L. Particle Mesh Ewald: An N·log(N) Method for Ewald Sums in Large Systems. *J. Chem. Phys.* **1993**, *98* (12), 10089–10092. https://doi.org/10.1063/1.464397.

(7) Wassenaar, T. A.; Ingólfsson, H. I.; Böckmann, R. A.; Tieleman, D. P.; Marrink, S. J. Computational Lipidomics with Insane: A Versatile Tool for Generating Custom Membranes for Molecular Simulations. *J. Chem. Theory Comput.* **2015**, *11* (5), 2144–2155. https://doi.org/10.1021/acs.jctc.5b00209.

(8) Martínez, L.; Andrade, R.; Birgin, E. G.; Martínez, J. M. PACKMOL: A Package for Building Initial Configurations for Molecular Dynamics Simulations. *J. Comput. Chem.* **2009**, *30* (13), 2157–2164. https://doi.org/10.1002/jcc.21224.

(9) Monticelli, L. On Atomistic and Coarse-Grained Models for C60 Fullerene. *J. Chem. Theory Comput.* **2012**, *8* (4), 1370–1378. https://doi.org/10.1021/ct3000102.

(10) Kästner, J. Umbrella Sampling. *WIREs Comput. Mol. Sci.* **2011**, *1* (6), 932–942. https://doi.org/10.1002/wcms.66.

(11) Ou, L.; Chen, H.; Yuan, B.; Yang, K. Membrane-Specific Binding of 4 Nm Lipid Nanoparticles Mediated by an Entropy-Driven Interaction Mechanism. *ACS Nano* **2022**, *16* (11), 18090–18100. https://doi.org/10.1021/acsnano.2c04774.

(12) Wei-Xin, X. U.; Yang, L. I.; H, Z. J. Z. Calculation of Collective Variable-Based PMF by Combining WHAM with Umbrella Sampling. *Chin Phys Lett* **2012**, *29* (6), 068702–068702. https://doi.org/10.1088/0256-307X/29/6/068702.

(13) Hub, J. S.; de Groot, B. L.; van der Spoel, D. G. g_wham—A Free Weighted Histogram Analysis Implementation Including Robust Error and Autocorrelation Estimates. *J. Chem. Theory Comput.* **2010**, *6* (12), 3713–3720. https://doi.org/10.1021/ct100494z.
